# Long-Term Enrichment of Stress-Tolerant Cellulolytic Soil Populations following Timber Harvesting Evidenced by Multi-Omic Stable Isotope Probing

**DOI:** 10.3389/fmicb.2017.00537

**Published:** 2017-04-11

**Authors:** Roland C. Wilhelm, Erick Cardenas, Hilary Leung, András Szeitz, Lionel D. Jensen, William W. Mohn

**Affiliations:** ^1^Department of Microbiology and Immunology, Life Sciences Institute, University of British ColumbiaVancouver, BC, Canada; ^2^Pharmaceutical Analytical Suite, Faculty of Pharmaceutical Sciences, University of British ColumbiaVancouver, BC, Canada

**Keywords:** timber harvesting, stable isotope probing, metagenomics, cellulose, decomposition, retention harvesting, disturbance ecology

## Abstract

Soil management is vital for maintaining the productivity of commercial forests, yet the long-term impact of timber harvesting on soil microbial communities remains largely a matter of conjecture. Decomposition of plant biomass, comprised mainly of lignocellulose, has a broad impact on nutrient cycling, microbial activity and physicochemical characteristics of soil. At “Long-term Soil Productivity Study” sites in California dominated by Ponderosa pine, we tested whether clear-cut timber harvesting, accompanied by varying degrees of organic matter (OM) removal, affected the activity and structure of the cellulose-degrading microbial populations 16 years after harvesting. Using a variety of experimental approaches, including stable isotope probing with ^13^C-labeled cellulose in soil microcosms, we demonstrated that harvesting led to a decrease in net respiration and cellulolytic activity. The decrease in cellulolytic activity was associated with an increased relative abundance of thermophilic, cellulolytic fungi (Chaetomiaceae), coupled with a decreased relative abundance of cellulolytic bacteria, particularly members of Opitutaceae, *Caulobacter*, and Streptomycetaceae. In general, harvesting led to an increase in stress-tolerant taxa (i.e., also non-cellulolytic taxa), though our results indicated that OM retention mitigated population shifts via buffering against abiotic changes. Stable-isotope probing improved shotgun metagenome assembly by 20-fold and enabled the recovery of 10 metagenome-assembled genomes of cellulolytic bacteria and fungi. Our study demonstrates the putative cellulolytic activity of a number of uncultured taxa and highlights the mineral soil layer as a reservoir of uncharacterized diversity of cellulose-degraders. It also and contributes to a growing body of research showing persistent changes in microbial community structure in the decades following forest harvesting.

## Introduction

The growing renewable energy economy presents new challenges for forest management from the increasing demand for lignocellulosic woody biomass previously left onsite (Allmér et al., 2009). One of the central concerns in forest management is whether harvesting affects soil nutrient capital and net primary productivity in the long-term, over multiple harvests (Keenan and Kimmins, 1993; Thiffault et al., 2011). In the interim between harvesting and full canopy closure of reforested land, soils experience substantial changes in the quantity and quality of organic matter (OM) input as well as greater exposure to solar radiation, higher averages and fluctuations in temperature (Kranabetter and Chapman, 1999; Kulmala et al., 2014) and lower near-surface moisture content (Childs and Flint, 1987; Adams et al., 1991; Paz, 2001; Redding et al., 2003). To understand the effects of these changes and improve forest soil management, the Long-Term Soil Productivity (LTSP) Study was initiated in 1989 as a longitudinal study of the impact of different OM removal on soil fertility (Powers et al., 2005), providing the experimental framework for this research.

The rate of decomposition influences a range of physicochemical properties of forest soils and has been reported to slow in the years following clear-cut harvesting in the short- (Whitford et al., 1981; Yin et al., 1989; Prescott et al., 2000; Fleming et al., 2006) and long-term (Holdena and Treseder, 2013; Webster et al., 2016). One major factor contributing to reduced rates of decomposition is a decrease in microbial biomass (Holdena and Treseder, 2013), yet changes in the composition of the decomposer community may also contribute. The loss of tree hosts and increase in belowground necrotic root tissue after clear-cutting can shift soil fungal communities from mycorrhiza-dominated to saprotroph-dominated systems (Hartmann et al., 2012). Changes in the structure of decomposer communities can also occur with reports of declining *Basidiomycota* and *Actinobacteria* populations and increases in *Ascomycota* (Bader et al., 1995; Hartmann et al., 2012; Štursová et al., 2014; McGuire et al., 2015). Differences in the lignocellulolytic capacity of soil communities between clear-cut and undisturbed forest plots has also been observed based on the carbohydrate-active gene content of metagenomes (Cardenas et al., 2015). This underlines the likelihood that compositional shifts have consequences for decomposition. Based on this array of evidence, we tested whether compositional changes in the active decomposer community could be observed by stable isotope probing (SIP) and whether we could identify a direct effect on the rate of decomposition.

SIP is commonly used to link microbial populations with functional activity in soils (Verastegui et al., 2014; Wang et al., 2015; Pepe-Ranney et al., 2016), but can also be used to establish whether changes in the rate of isotope assimilation correspond with shifts in functional populations. To test for the long-term effects of timber harvesting on soil decomposer populations, we performed SIP using ^13^C-labeled cellulose which comprises the greatest form of carbon (~45%) in coniferous, softwood tree biomass (Keijsers et al., 2013). We expect that the warmer, drier, near-surface soil conditions in harvested plots would select for unique cellulolytic populations such as dark-septate fungi (Gallo et al., 2009) and cellulolytic bacteria adapted to hot and arid conditions (Rastogi et al., 2010; Gabani et al., 2012; Soares et al., 2012). We also expect that long-term changes in the quality and quantity of litter inputs may drive differences in cellulolytic populations, in particular in harvested plots where coarse woody debris was retained. Ascomycota are known to predominate on younger forms of detritus compared to Basidiomycota, which succeed in later stages of decomposition (Edwards et al., 2011; Voriskova and Baldrian, 2013). We used a ^13^C-labeled cellulose to determine whether long-term changes in environmental conditions and OM removal affect the composition of specifically cellulolytic populations and the rate of cellulose decomposition.

To date, there has been one SIP-based investigation into the effects of forest disturbance (prescribed burning) on cellulolytic communities, but this study (Bastias et al., 2009), along with other recent cellulose-based SIP research (Schellenberger et al., 2010; Štursová et al., 2012; Koranda et al., 2014; Torres et al., 2014; Kramer et al., 2016), utilized commercially available ^13^C-cellulose of low purity according to the manufacturer (58% glucose, 4.4% lignin, unknown percentage of sugars from hemicellulose; see Supplementary Data 1), raising the possibility that a substantial proportion of reported carbon assimilation was not from cellulose. In the present study, we employed a much purer (99%) form of bacterial ^13^C-labeled cellulose. Bacterial cellulose has similar mechanical properties to plant cell walls, particularly in traits correlated to enzymatic degradation, which include comparable polymer length (3,000–9,000 units) and crystallinity (80–90% crystalline) (Chanliaud et al., 2002). Bacterial cellulose was previously used in SIP applications (El Zahar Haichar et al., 2007; Pinnell et al., 2014).

**Figure 1:**
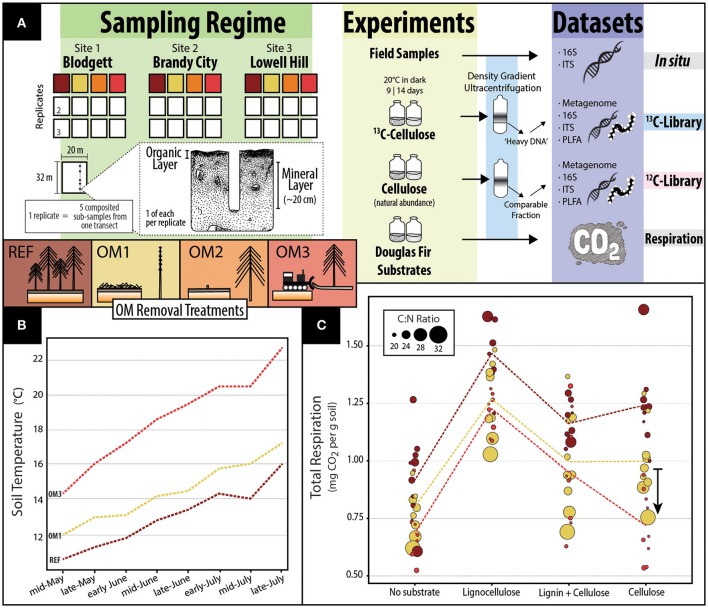
**A composite figure providing (A)** an overview of the sampling, experiments and datasets in this study; **(B)** soil temperatures in summer averaged across all sites and the entire soil profile for REF, OM1, and OM3 5 years after harvesting (sourced from Paz, 2001 and reprinted with permission from Dr. Lucas Paz); and, **(C)** a dot-plot showing soil respiration in microcosms with mineral soils. In **(C)**, the colored lines represent average values of each treatment (*n* = 9). Dot area is scaled to carbon to nitrogen ratio of individual soil samples. An arrow depicts the interaction between OM3 and respiration with cellulose, which was statistically supported [*t*_(11, 94)_ = −2.65; *p* = 0.01].

Sampling was conducted at three LTSP sites in California 16 years after harvest and replanting. The activity and composition of cellulolytic populations were characterized using a multi-omic SIP approach that included quantitative measurements of respiration and ^13^C-enrichment of phospholipid fatty acids (PLFA), along with relative abundance data that included SIP-shotgun metagenomes, SIP-pyrotag 16S rRNA gene and ITS region amplicon libraries (overview in Figure 1A). This data collection forms part of the LTSP's aim to identify indicators of soil quality and soil process relevant to monitoring forest regeneration. In addition to testing the impacts of timber harvesting, we sought to expand general knowledge of cellulolytic populations in forest soils and to characterize the cellulolytic potential of mineral layer soils for the first time. We provide one of the first examples of SIP coupled to shotgun metagenomics (others include Grob et al., 2015), for which we describe the effective enrichment and recovery of metagenome-assembled genomes (MAGs) from novel, uncultured cellulolytic taxa.

## Materials and methods

### Sampling sites and sample collection

Soil samples were collected from three sites (Blodgett, Brandy City, and Lowell Hill) in the Sierra Nevada of California which were harvested and reforested with ponderosa pine 16 years previously as part of the Cohasset soil series of the Long-Term Soil Productivity Study (Powers, 2006). The distance between sites ranged from 20 to 57 km and all shared similar forest cover and soil type (Mesic Ultic Haploxeralfs). Four treatments were sampled at each site: an unharvested reference plot (REF) and three harvested treatments accompanied by varying degrees of OM removal. Harvested treatments consisted of OM1, where tree stems (trunks) were removed, but branches and woody debris were retained; OM2, where whole tree biomass was removed; and OM3, where whole tree biomass plus the upper organic layer of the soil were removed. Photographs of harvested treatments are displayed in Figure S1. Triplicate samples were taken at each plot (45 m^2^) each comprised of five sub-sampled points along a plot transect to account for heterogeneity and ensure sufficient soil material. In sampling, the litter layer was removed from vegetation-free soil and the organic layer (the O-horizon) was collected with a trowel. Next, the top 20 cm of mineral soil (including the A and occasionally upper B-horizon) was collected using a Stoney auger (5 cm diameter). Samples were stored at −80°C and processed within 1 year. For an overview of sample collection and the experimental design consult Figure 1A.

### Soil respiration assays

Organic and mineral layer soil samples from REF, OM1 and OM3 were incubated in microcosms with no additional substrate or with one of three milled lignocellulosic substrates derived from Douglas-fir: (i) “lignocellulose,” from debarked, untreated Douglas-fir woodchips, (ii) “lignin + cellulose,” from steam treated woodchips, where hemicellulose was solubilized and removed, and (iii) “cellulose,” from steam treated woodchips which were subsequently delignified (Kumar et al., 2012). Microcosms were prepared by adding 4.5 g dry wt soil to 30-mL serum vials, adjusting moisture content to 60% (mineral) and 125% (organic) (w/v) and pre-incubated at 20°C for 1 week. Substrate was then added (10% w/w) along with CO_2_ traps, consisting of sterile glass vials containing 2 mL NaOH (1M). Microcosms were then incubated at 20°C for 14 days based on time course experiments described in Wilhelm et al. (2014). Net respiration was determined by titration of the NaOH traps according to methods described by Haney et al. (2008). OM2 samples were not tested here, and in small number of other experiments, due to limitations in the quantity of available substrate.

### SIP microcosms

Soil from all samples (*n* = 72) was incubated in paired treatments: one amended with 10% *w/w* of ^13^C-labeled cellulose (99 atom % ^13^C) and another with the same amount of unlabeled cellulose (natural abundance ^13^C: ~1%). The ^12^C-control incubations were included to correct for natural ^13^C content in SIP-phospholipid fatty acid (PLFA) work and to control for native populations with higher GC content (i.e., slightly heavier DNA) in SIP-DNA work, as described below. Bacterial cellulose (>99% glucose) was produced by feeding *Gluconacetobacter xylinus* with ^13^C-labeled glucose (see Supplementary Data 5, for details). Microcosm preparation was identical to previously described respiration assays, except for the following differences: 1.5 g (organic) and 2 g (mineral) dry wt soil were used, and incubations were for 11 days (organic) and 14 days (mineral). Following incubation, soil was lyophilized and stored at −80°C until processing. All SIP-PLFA, SIP-pyrotag and SIP-metagenomic data were derived from the same set of microcosms. Libraries termed “*in situ*” were derived from corresponding field soil samples that were not incubated and, post-hoc, from publicly available pyrotag libraries from LTSP sites in British Columbia (Hartmann et al., 2012).

### SIP phospholipid fatty acids (PLFA)

PLFAs were extracted according to Bligh and Dyer (1959) and the ^13^C-content was analyzed using IRMS (University of British Columbia Stable Isotope Facility) ported with gas chromatography as detailed in Churchland et al. (2013). Peak identification was based on retention time compared against two reference standards: the bacterial acid methyl-ester standard (47080-0; Sigma–Aldrich, St. Louis) and a 37-Component fatty acid methyl-ester mix (47885-U; Sigma–Aldrich, St. Louis). Unidentifiable ^13^C-enriched peaks were also included in analysis if they met the following conditions: (i) detection in 3 or more samples, (ii) average δ ^13^C > +50%0 and (iii) confirmed as long-chain alkane methyl esters by GC-MS. Taxonomic affiliations of specific PLFAs were assigned according to Högberg et al. (2013), with c18:1ω9 and c18:3ω6 added as additional fungal PLFAs (Ruess and Chamberlain, 2010).

### SIP DNA-pyrosequencing and metagenomic library preparation

DNA was extracted from soil (0.5 g) with the manufacturer's recommended protocol for the FastDNA™ Spin Kit for Soil (MPBio, Santa Ana, CA). The mass and the atom % ^13^C of DNA extracts were measured with UHPLC-MS/MS according to Wilhelm et al. (2014). DNA extracts from replicates within each site were pooled in equal amounts and unlabeled controls were processed identically (*n* = 24 × 2). ^13^C-enriched DNA was recovered by density gradient ultracentrifugation according to methods in Neufeld et al. (2007) and Wilhelm et al. (2014), with improvements for greater recovery of DNA (see Supplementary Data 5). Both SIP and *in situ* pyrotag libraries were prepared from the 16S rRNA gene (V1–V3 regions) as well as fungal internal transcribed spacer region (ITS2) according to the procedure of Hartmann et al. (2012). Metagenomic libraries were prepared from 40–50 ng of enriched DNA using the Nextera DNA Sample Preparation Kit (Illumina Inc., CA, USA). Four shotgun metagenome libraries were generated, ^13^C-libraries from REF, OM1 and OM3 treatments as well as a ^12^C-library from the REF treatment, by pooling the corresponding DNA extracts from mineral layer samples at all three sites. These four libraries were multiplexed on two lanes of Illumina HiSeq (2 × 100-bp), yielding 285 million paired-end reads. There was insufficient ^13^C-enriched DNA to generate metagenomes for the organic layer samples.

### Statistical and bioinformatic analysis

Statistical analyses were performed using the R platform (v. 3.1.0; R Core Team, 2015). 16S rRNA gene libraries were quality-filtered and processed using Mothur (Schloss et al., 2009) according to the Schloss “454 SOP” (http://www.mothur.org/wiki/454_SOP; accessed May 2013) and were clustered into operational taxonomic units (OTUs) at 0.01% dissimilarity. ITS libraries were processed according to Hartmann et al. (2012) to create OTUs, but, due to the hypervariability of the ITS region, were also grouped based on taxonomic classification using UNITE (Kõljalg et al., 2013). We used three methods to identify OTUs differentially abundant between ^12^C-control and ^13^C-enriched library samples: “DESeq” (Anders and Huber, 2010), “limma-voom” (Ritchie et al., 2015) and uncorrected, averaged relative abundance. An OTU was deemed ^13^C-enriched if it had at least a 3-fold higher relative abundance in ^13^C vs. ^12^C libraries according to one or more of the methods. The identification of carbohydrate-active enzyme (CAZy') genes was based on BLASTX searches using methods in Cardenas et al. (2015). The following glycosyl hydrolase (GH) families contain enzymes with endoglucanase activity: GH5, 6, 7, 8, 9, 12, 16, 44, 45, 48, 51, 61, 74, 81, and 131. Shotgun metagenome libraries were preprocessed using Trimmomatic (Bolger et al., 2014; v. 0.32), to trim sequencing primers and low quality ends, and the FastX Toolkit (Gordon and Hannon, 2010; v. 0.7), to filter short or low quality reads. Paired-end and orphaned reads were all assembled using the default setting of Ray-meta (kmer size = 39 bp) (Boisvert et al., 2012; v. 2.3.1). Subsequent binning of contigs into putative genome bins was performed with Metawatt (Strous et al., 2012; v. 2.1), based on tetranucleotide frequency, and MetaBAT (Kang et al., 2014; v. 0.18.6), based on both tetranucleotide frequency and covariance in read abundance mapped to the super assembly. The completeness of genome bins was assessed by scanning for essential single-copy, house-keeping genes with hidden Markov models provided by Albertsen et al. (2013). Taxonomic designations were based on lowest-common ancestor analysis via MEGAN using matches to the NCBI “nr” database (v. 5.10.1; Huson et al., 2007). Reads from metagenomes were mapped back to genome bins using Bowtie2 (Langmead and Salzberg, 2012) to estimate their relative abundances among harvested treatments. Additional details can be found in Supplementary Data 5, while a script for all R analyses and raw data can be found in Supplementary Data 2.

### Data accessibility

Raw sequence data were deposited at the European Nucleotide Archive under the study accession (PRJEB9761) for 16S rRNA gene pyrotags (ERS803692-ERS803739) ITS pyrotags (ERS803740-ERS803786), binned genomes (ERZ288956 - ERZ288966), and metagenomic libraries (ERS1099581- ERS1099584). Raw data used in all other analysis, namely soil chemistry data, net respiration, PLFA, CAZyme abundances (among others) can be found in Supplementary Data 2.

## Results

### Harvesting effects on decomposer activity

Comparisons of decomposer activity were based on net respiration and total ^13^C assimilated from cellulose into PLFA and DNA. Net respiration was significantly lower in mineral soil microcosms from harvested plots, OM1 [*t*_(2, 103)_ = −3.18, *p* < 0.001] and OM3 [*t*_(2, 103)_ = −4.99, *p* < 0.001], relative to reference plots (REF), with or without amendment of Douglas-fir lignocellulosic substrates (Figure 1C). OM1 soil had the highest carbon content (Table 1), consistent with the retention of woody debris, yet produced less CO_2_ than REF even though carbon content was weakly correlated with respiration (Spearman's *r* = 0.19; *p* = 0.049). Respiration in cellulose-amended soil from OM3 was particularly low, suggesting that cellulose-degrading populations were disproportionately affected by the greatest degree of OM removal [*t*_(11, 94)_ = −2.65; *p* < 0.01; arrow in Figure 1C]. Respiration was also positively correlated with pH (Spearman's *r* = 0.34; *p* < 0.001), which was lowest in OM1, but not with total ^12^C PLFA (Spearman's *r* = 0.05; *p* = 0.82), total nitrogen (Spearman's *r* = 0.18; *p* = 0.07) or C:N ratio (Spearman's *r* = −0.07; *p* = 0.45). Organic layer soils respired 3-fold more CO_2_ than mineral soils, but no significant differences among OM removal treatments were observed. Across all experiments, measurements of organic layer soils were highly variable. In respiration assays, differences in respiration even among organic soil microcosms with or without added substrate were obscured by this variability.

**Table 1:** **Soil properties and microbial activity in microcosms incubated with ^**13**^C-cellulose**.

		**Organic layer**				**Mineral layer**			
		**REF**	**OM1**	**OM2**	**OM3**	**REF**	**OM1**	**OM2**	**OM3**
Soil compositio*n* (*n* = 9)	Average Percent Carbon	37.0 ± 2.2	41.5^a^ ± 1.0	33.0 ± 2.4	31.5^b^ ± 3.6	5.9 ± 0.5	7.6 ± 0.7	−	6.4 ± 0.8
	Average Percent Nitrogen	1.21 ± 0.09	1.34 ± 0.11	1.21 ± 0.13	1.10 ± 0.13	0.26 ± 0.02	0.31 ± 0.04	−	0.32 ± 0.04
	Average C:N Ratio	31.1 ± 1.5	32.5 ± 3.3	28.2 ± 1.7	29.5 ± 2.2	22.3 ± 0.9	25.4^a^ ± 1.3	−	20.0^b^ ± .03
	Average pH	5.47 ± 0.2	4.35^a^ ± 0.2	5.1 ± 0.2	5.2^b^ ± 0.1	6.1 ± 0.2	5.5 ± 0.1	−	5.6 ± 0.1
Repiration (*n* = 36)	Average mg CO_2_ per g soil	−	−	−	−	1.20^a^ ± 0.04	1.01^b^ ± 0.04	−	0.90^c^ ± 0.05
PLFA Biomass Measures (*n* = 9)	Average Delta ^13^C	1, 600 ± 110	1, 100 ± 130	2, 300 ± 170	1, 400 ± 70	6, 400^a^ ± 870	4, 800 ± 650	4, 800 ± 700	3, 200^b^ ± 540
	Total ^13^C carbon (μmol ^13^C per g soil)	0.96^a^	0.45^b^	0.62	0.68	0.42^a^	0.43^a^	0.41^a^	0.24^b^
	Total ^12^C carbon (μmol ^12^C per g soil)	32.2^a^	19.0^b^	16.6^b^	25.8^a^	5.0^a^	6.6^b^	6.2	5.0
	Median number of enriched FA	33^a^ ± 0.9	27^c^ ± 1.2	27^b^ ± 0.7	25^c^ ± 0.5	29 ± 1.2	29 ± 0.7	29 ± 0.6	27 ± 0.7
	Fungal:Bacteria Ratio	0.78^a^ ± 0.07	0.95^a^ ± 0.05	1.68^b^ ± 0.21	1.95^b^ ± 0.48	0.64 ± 0.13	0.71 ± 0.15	0.92 ± 0.13	1.03 ± 0.23
DNA enrichment (*n* = 3)	Atom % ^13^C above natural abundance	4.8 ± 1.0	4.5 ± 1.5	5.6 ± 1.1	3.3 ± 0.5	14.6^a^ ± 2.2	9.9 ± 1.1	10.3 ± 0.6	9.1^b^ ± 1.1

*Values denoted by different letters are significantly different (p < 0.05) based on Tukey's Honest Significant Difference. Respiration data from organic layer soils was not included because of extreme variability. Technical error (S.E.) for δ-^13^C is approximately 80‰, equivalent to 3 × 10^−4^ μmol C*.

**Figure 2:**
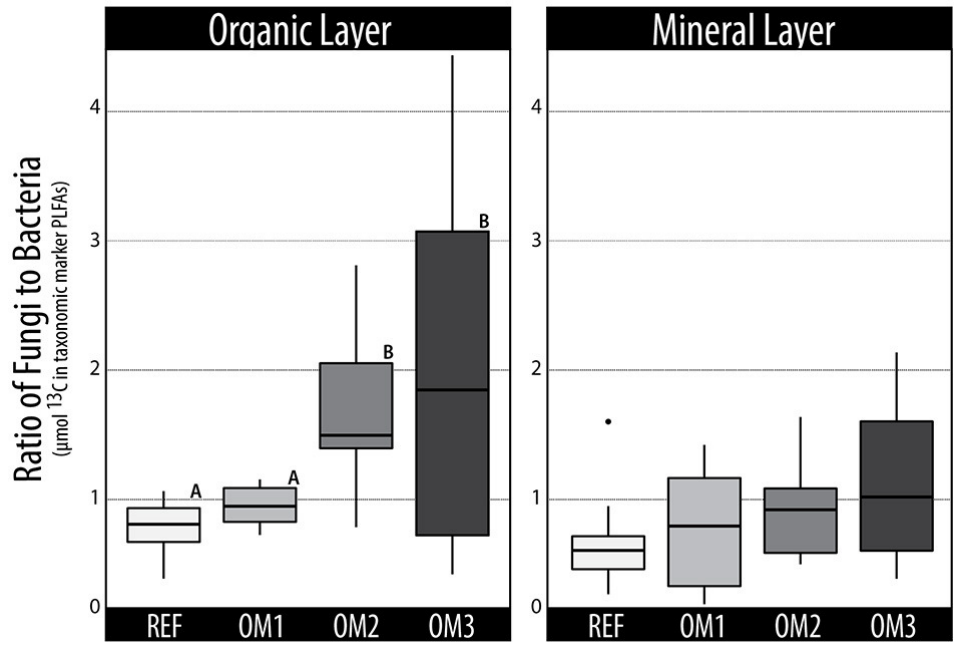
**Trends in the total ^**13**^C-enrichment of fungal vs. bacterial PLFAs in organic and mineral layer soils**. Statistically supported differences (TukeyHSD; *p* < 0.01) are grouped by lettering.

The incorporation of ^13^C into PLFAs and DNA was generally lower in soils from harvested plots, with the clearest evidence occurring in mineral soils (Table 1). The total assimilation by bacteria and fungi also differed among harvested treatments, with significantly greater bacterial assimilation in both REF and OM1 (Figure 2). The differences in bacterial and fungal activity was evident in both ^12^C and ^13^C PLFA profiles, driven by an increase in fungal biomass in OM3 (1.8-fold higher than REF) relative to Gram-positive and Gram-negative bacterial biomass in REF, 1.4-fold and 1.3-fold higher than OM3, respectively. The proportion of ^13^C-enriched Gram-positive PLFAs was highest in REF (47%) followed by OM1 (35%), OM2 (31%), and OM3 (28%). Overall, the organic layer contained 4-fold greater microbial biomass, based on total PLFAs, and exhibited greater total cellulolytic activity. Minor differences in cellulolytic activity were observed among sites, which corresponded to differences in total biomass (Table S1).

### Harvesting effects on community structure

**Figure 3:**
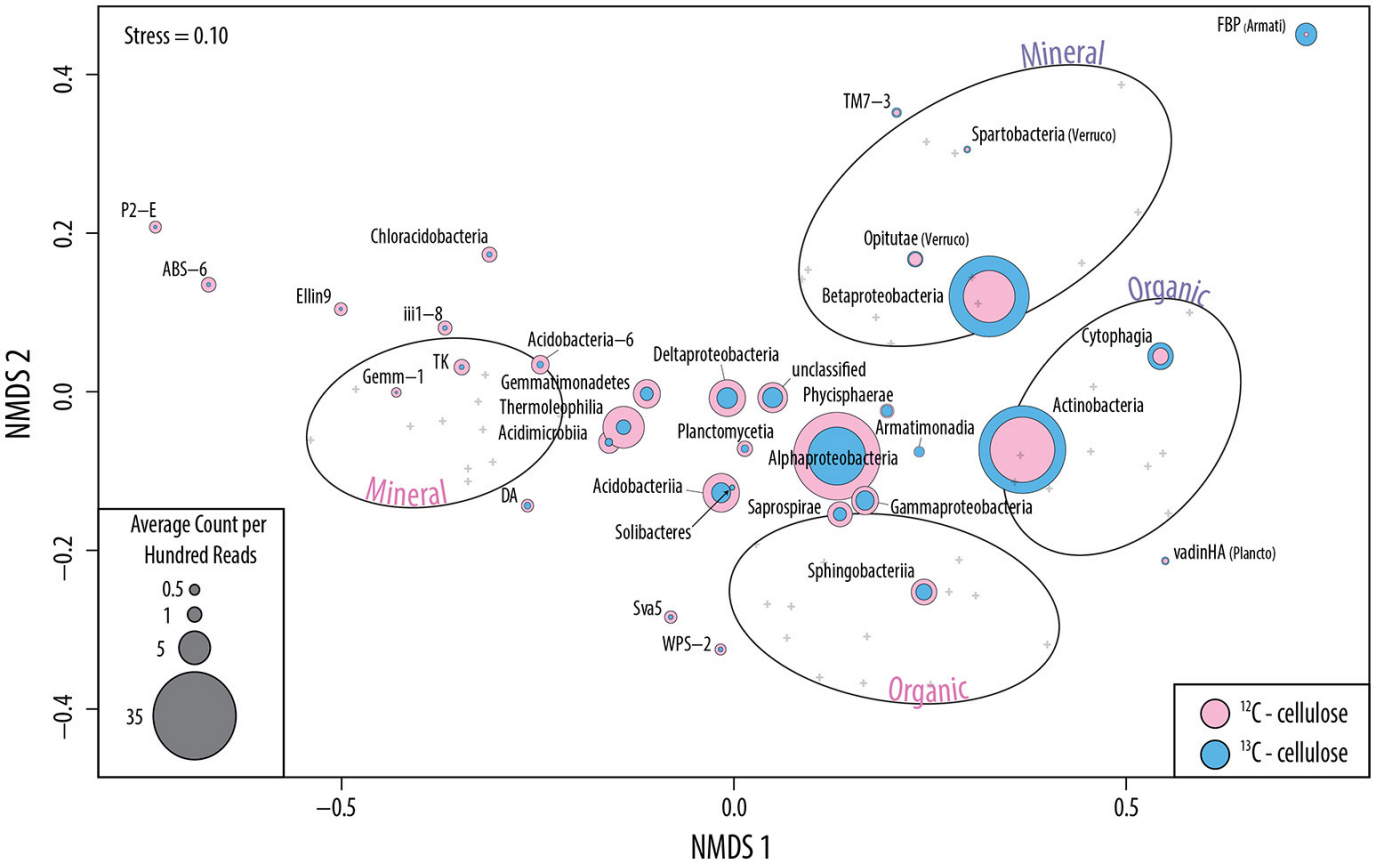
**Non-metric multidimensional scaling of 16S rRNA gene pyrotag libraries based on Bray-Curtis dissimilarities**. Ovals indicate the 95% confidence interval for the distribution of samples which are denoted by gray crosses. Colored circles represent the ordination of bacterial classes of greater than 0.15% overall relative abundance and are scaled to their normalized abundances in ^12^C- (pink) and ^13^C-libraries (blue). Candidate taxa without designated classes are identified as FBP (division of Armatimonadetes) and WPS-2 (phylum).

The successful targeting of cellulolytic populations in sequencing libraries via SIP was supported by the following: (i) significantly higher quantities of ^13^C in soil DNA extracts and a corresponding 2.5-fold higher concentration of DNA in heavy CsCl gradient fractions (Figure S2); (ii) distinct clustering of samples according to ^13^C-enrichment and soil layer in NMDS ordinations (Figure 3); (iii) the vastly improved assembly of metagenomes from ^13^C-enriched DNA (~20%) relative to the control library (<1%) (Table S2) and (iv) lower alpha-diversity of all ^13^C-libraries relative to ^12^C- and *in situ* libraries (Figure S3). Harvested treatments accounted for ~9% of the total variation in ^13^C-pyrotag OTU profiles (perMANOVA; *F* = 1.3; *p* = 0.04), which was comparable to the amount explained by soil layer (Figure S4). Harvesting was not a significant factor in explaining differences among fungal ^13^C-pyrotag profiles. Harvesting did not produce differences in alpha diversity (Shannon-Wiener diversity; Figure S3) or beta diversity (UniFrac) of ^13^C-pyrotag libraries, suggesting no substantial loss or gain of cellulolytic groups occurred. However, harvesting did alter the relative abundance of major taxa incorporating ^13^C from cellulose during incubations.

**Figure 4:**
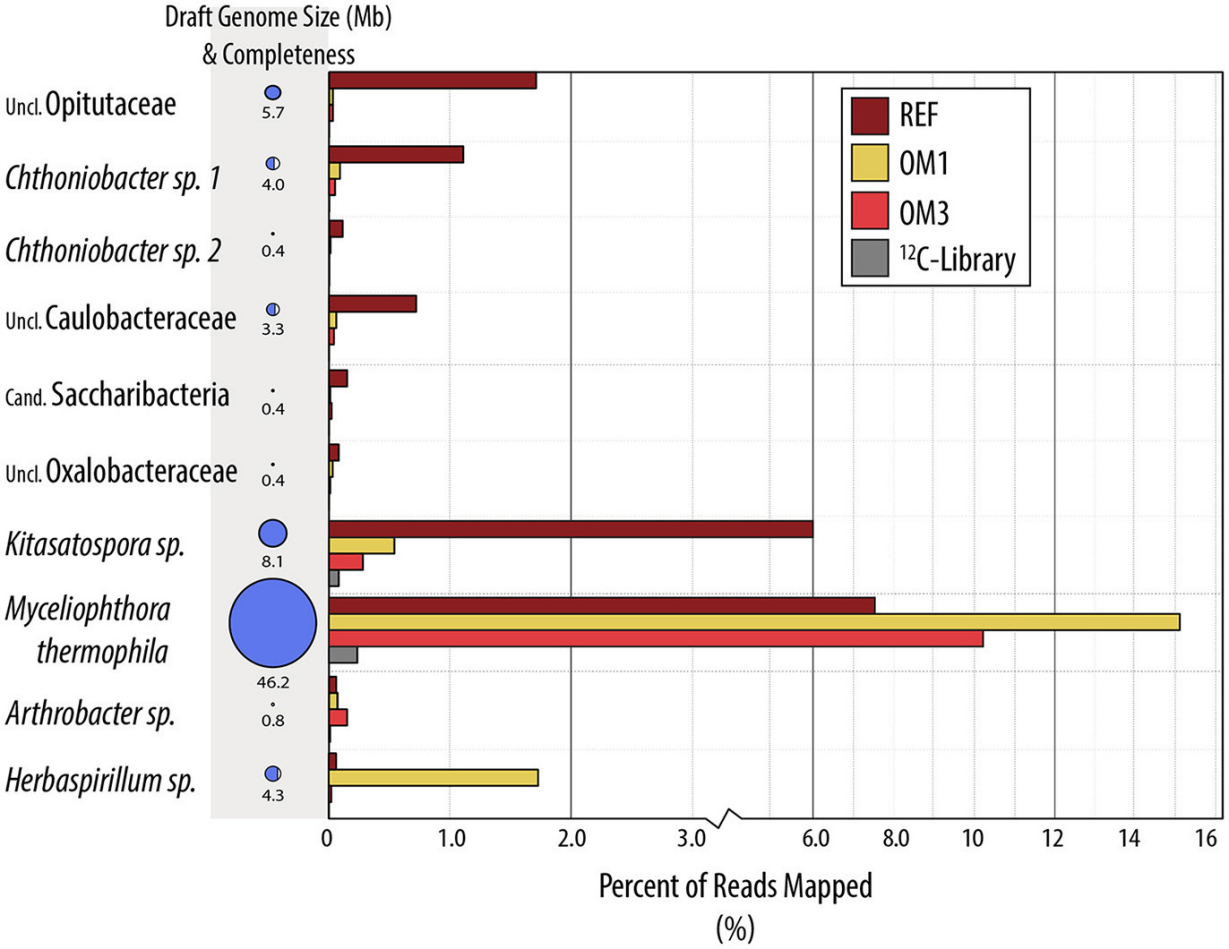
**Draft genome bins recovered from metagenome assemblies from ^**13**^C-enriched DNA**. Bars indicate the percentage of reads contributed by metagenomes from each treatment group. Genome size corresponds to size of bubble (also written) and completeness to the bubble fill. For additional details on completeness, taxonomic uniformity and accession numbers, consult Table S3.

**Figure 5:**
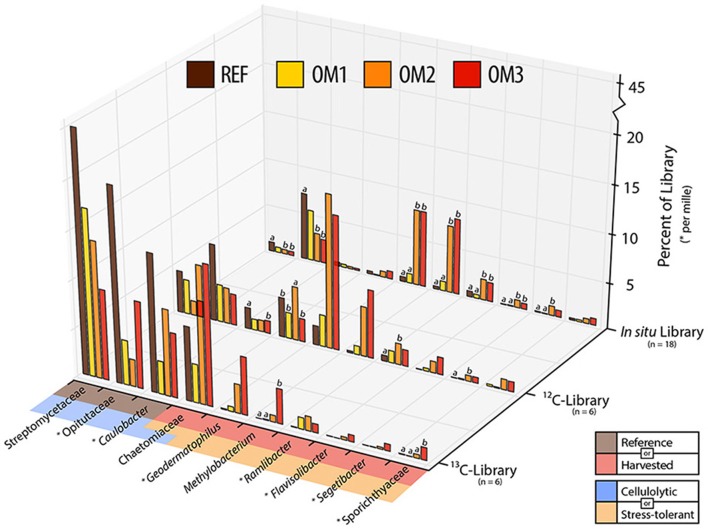
**The relative abundance of indicators of either reference (beige) or harvested (red) treatments in ^**13**^C-, ^**12**^C- or ***in situ*** (i.e., field samples) 16S rRNA gene or ITS pyrotag libraries**. Taxa were designated as cellulolytic (blue), in this study, and/or previously reported to be desiccation and/or heat tolerant (pink). Abundances of taxa with asterix (^*^) represent per mil, rather than per cent abundance. Counts are combined from organic and mineral soil layers and trends were apparent in both layers. Statistically supported differences (TukeyHSD; *p* < 0.01) are grouped by lettering.

**Figure 6:**
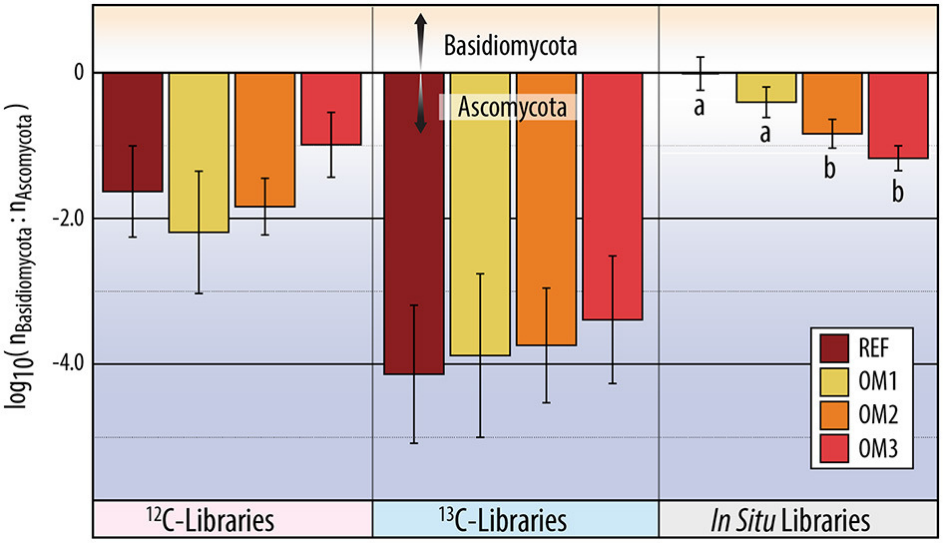
**The ratio of total reads classified to Basidiomycota vs. Ascomycota in ITS pyrotag libraries**. The y-axis corresponds to the log of the ratio of Basidiomycota to Ascomycota. Statistically supported differences (TukeyHSD; *p* < 0.01) are grouped by lettering.

^13^C-pyrotag libraries from harvested plots had diminished relative abundances of putatively cellulolytic Verrucomicrobia (*Chthoniobacter* and unclassified Opitutaceae), uncl. Streptomycetaceae, *Burkholderia*, uncl. Rhizobiaceae and *Caulobacter* (Table 2). Populations of Verrucomicrobia, Streptomycetaceae (*Kitasatospora sp*.) and Caulobacteraceae were sufficiently abundant and active (i.e., differentially abundant in ^13^C-libraries) to recover sizeable metagenome-assembled genomes (MAGs) (Table S3). Further evidence for the contraction of these populations as a result of harvesting was found in the reduced proportion of metagenomic reads which mapped to their MAGs (Figure 4) as well as their reduced relative abundances in ^12^C- and *in situ* pyrotag libraries (Figure 5). Conversely, a number of putatively cellulolytic taxa increased in abundance in soils from harvested plots, including a number of Betaproteobacteria and members of Myxococcales, Planctomycetes and fungi belonging to the ascomycotal family Chaetomiaceae and basidiomycotal genus *Clitopilus* (Table 2). The active member of Chaetomiacae was also sufficiently abundant and active to recover its 43-Mb MAG (Table S3). Overall, Ascomycota were between ~10^3^ and 10^4^ times more abundant than Basidiomycota in ^13^C-pyrotag libraries. The ratio of Basidiomycota to Ascomycota did not significantly differ among harvested plots in either ^12^C- or ^13^C-libraries; however, the ratio did significantly decrease *in situ* in OM2 [*t*_(3, 65)_ = −2.97; *p* < 0.01] and OM3 [*t*_(3, 65)_ = −4.18; *p* < 0.01] relative to REF (Figure 6).

**Table 2:**
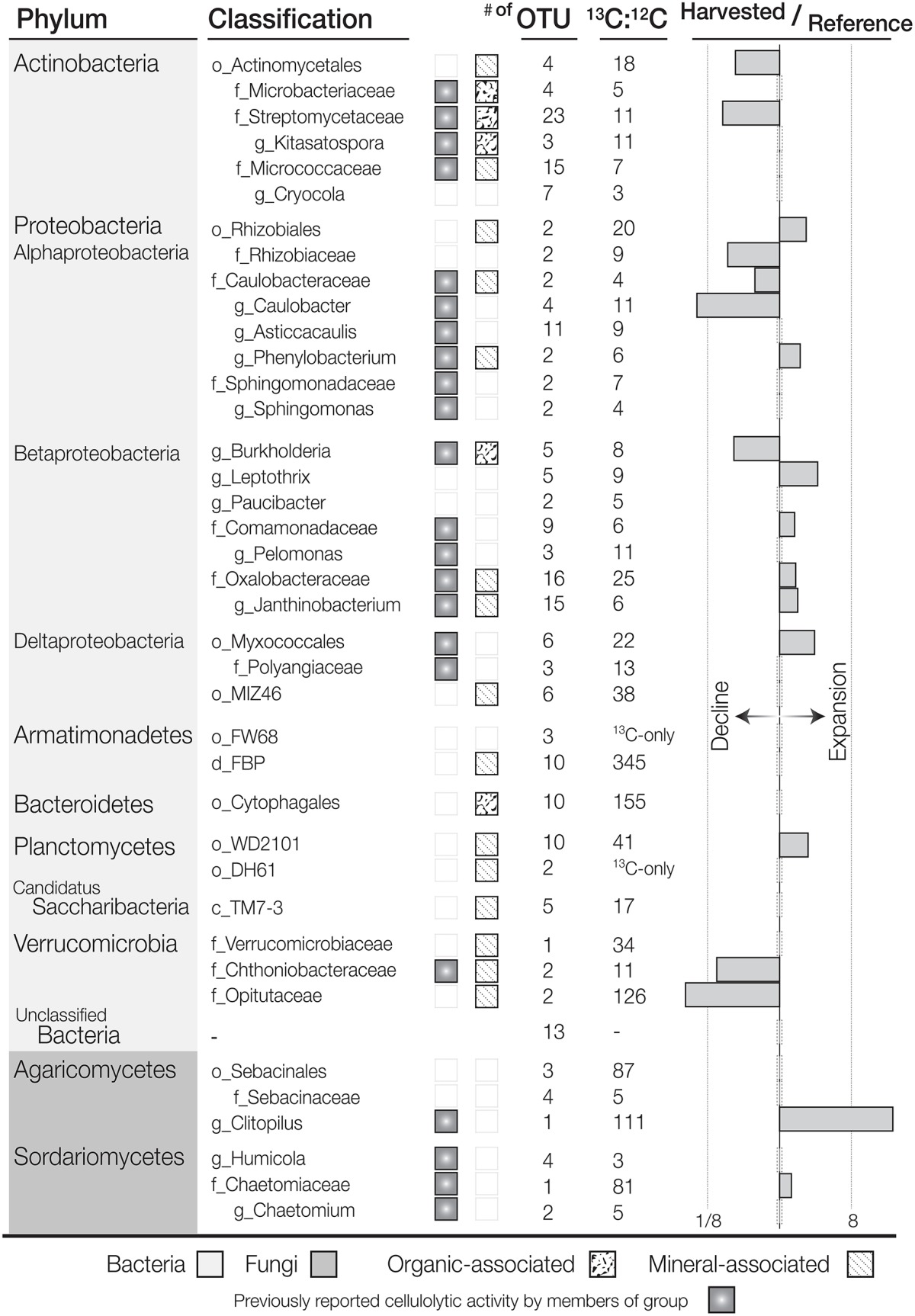
**List of putatively cellulolytic bacterial and fungal taxa determined by differential abundance between ^**13**^C- and ^**12**^C-16S rRNA or ITS pyrotag libraries (^**13**^C:^**12**^C)**.

*“Harvested/Reference” depicts all taxa significantly (p < 0.05) more abundant in microcosms with soil from harvested plots or reference plots based on log response ratio (the natural log of the mean abundance in soil from harvested plots divided by the mean abundance in soil from reference plots). Mineral layer and organic layer-associations are denoted by patterned squares. Taxa with previously reported cellulolytic activity are denoted by solid circles. Classification refers to the lowest possible taxonomic rank for the group of OTUs (bootstrap > 80), meaning they were unclassified at lower taxonomic ranks. Each classification is prefaced with its associated rank (i.e., “o__” corresponds to “order,” etc.). The “# enrOTU” represents the total number of ^13^C-enriched OTUs assigned to a taxon. A full list of enrOTUs with corresponding sequence accession numbers can be found in Supplementary Data 4*.

**Figure 7:**
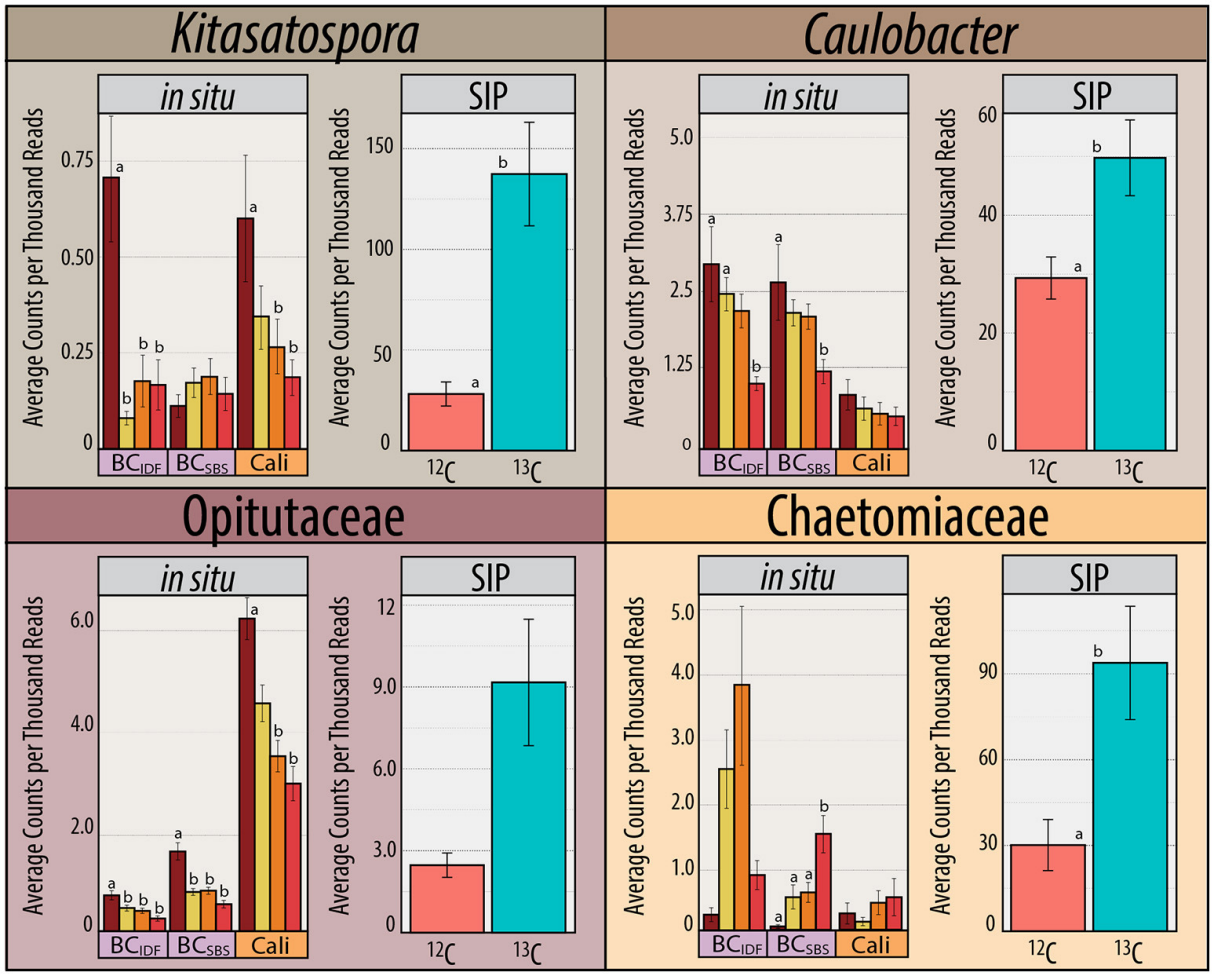
**The ***in situ*** relative abundance of ***Kitasatospora*** (Streptomycetaceae), ***Caulobacter***, Opitutaceae, and Chaetomiaceae in pyrotag libraries from field sites in California and two LTSP field sites in British Columbia (IDF and SBS) previously published by Hartmann et al. (**2012**)**. Accompanying relative abundances in ^13^C- vs. ^12^C-pyrotag libraries from SIP-microcosms with California soil (pooled REF and OM samples) are provided. Counts are combined from organic and mineral soil layers and trends were apparent in both layers.

The increased ratio of fungi to Gram-positive bacteria in harvested treatments, observed in PLFA data, was corroborated by similar changes in the relative abundance of Streptomycetaceae (Actinobacteria) and Chaetomiaceae (Ascomycota) in pyrotag libraries and shotgun metagenomes. The trend was apparent in both ^13^C- and *in situ* pyrotag libraries (Figure 5) as well as by read mapping to the *Kitasatospora* (Streptomycetaceae) and *Myceliophthora thermophila* MAGs (Chaetomiaceae) (Figure 4). The decline of Streptomycetaceae (*Kitasatospora*), along with *Caulobacter* and Opitutaceae, and increase in relative abundance of Chaetomiaceae was corroborated by previously published pyrotag libraries from LTSP field sites in British Columbia (Figure 7). Chaetomiaceae were highly abundant in all ITS libraries in the present study, comprising ~0.5, 3, and 9% of total *in situ*, ^12^-C and ^13^C-libraries. Both Sordariomycetes (Spearman's *r* = −0.37, *p* = 0.06) and Actinobacteria (*r* = −0.39, *p* = 0.05) were negatively correlated with C:N ratio, while Sordariomycetes were positively (*r* = 0.42, *p* = 0.03) and Actinobacteria negatively (*r* = −0.38, *p* = 0.05) correlated with total carbon in mineral layer soil (*in situ* libraries). The relative abundance of the aforementioned taxa did not significantly differ among the three sites (Blodgett, Brandy City, and Lowell Hill), while some taxa exhibited soil layer preferences (Table 2).

Indicator species analysis identified bacterial taxa with consistently increased relative abundance in soils from harvested plots across all pyrotag libraries (Figure 5; Supplementary Data 3). All of these taxa have members who are reported to be tolerant of heat, radiation and desiccation: *Geodermatophilus* (Montero-Calasanz et al., 2014; Sghaier et al., 2016), *Sporichthya* (Eppard et al., 1996; Babalola et al., 2009), *Ramlibacter* (De Luca et al., 2011), *Flavisolibacter* (Joo et al., 2015), *Methylobacterium* (Nogueira et al., 1998; Rokitko et al., 2003) and *Segetibacter* (Liu et al., 2014). The relative abundance of these groups (except *Flavisolibacter* and Sporichthyaceae) was also substantially increased in harvested plots from LTSP field sites in British Columbia (Figure S5).

### Cellulolytic taxa

A total of 234 bacterial ^13^C-enriched OTUs (enrOTUs) were identified, representing nine phyla. EnrOTUs classified as Actinomycetales, Armatimonadetes, Cytophagales, Myxococcales, Planctomycetes, Rhizobiales, Opitutaceae and Oxalobacteraceae were the most highly enriched (Table 2). Non-metric multidimensional scaling confirmed broad differences between ^13^C- and ^12^C-pyrotag libraries as well as distinct cellulolytic bacterial populations in each soil layer (Figure 3). EnrOTU from the organic-rich soil layer were mainly represented by previously known cellulose-degrading phyla, Cytophaga and Actinobacteria, while the mineral layer contained Betaproteobacteria and less characterized phyla such as Armatimonadetes (candidate division FBP and order FW68), Verrucomicrobia (classes Opitutae and Spartobacteria) and candidatus Saccharibacteria (formerly TM7). The delineation of fungal enrOTUs was less successful due to sparse overlap amongst OTUs in ITS libraries, which were typically dominated by few, highly abundant OTUs. This was illustrated by the poor separation of samples by NMDS (Figure S6) and the relatively small number of fungal enrOTUs identified (*n* = 16). These enrOTUs included unclassified Ascomycota and members of Agaricomycetes and Sordariomycetes (Table 2), while clustering in NMDS suggested the involvement of Dothideomycetes and a large proportion of unclassified ITS sequences. Ascomycota were major cellulose degraders under our experimental conditions as evidenced by the massive difference between ^13^C- vs. ^12^C- metagenomes in the proportion of reads classified as Ascomycota (an average of 10.6 and 0.8%, respectively; Figure S7).

**Figure 8:**
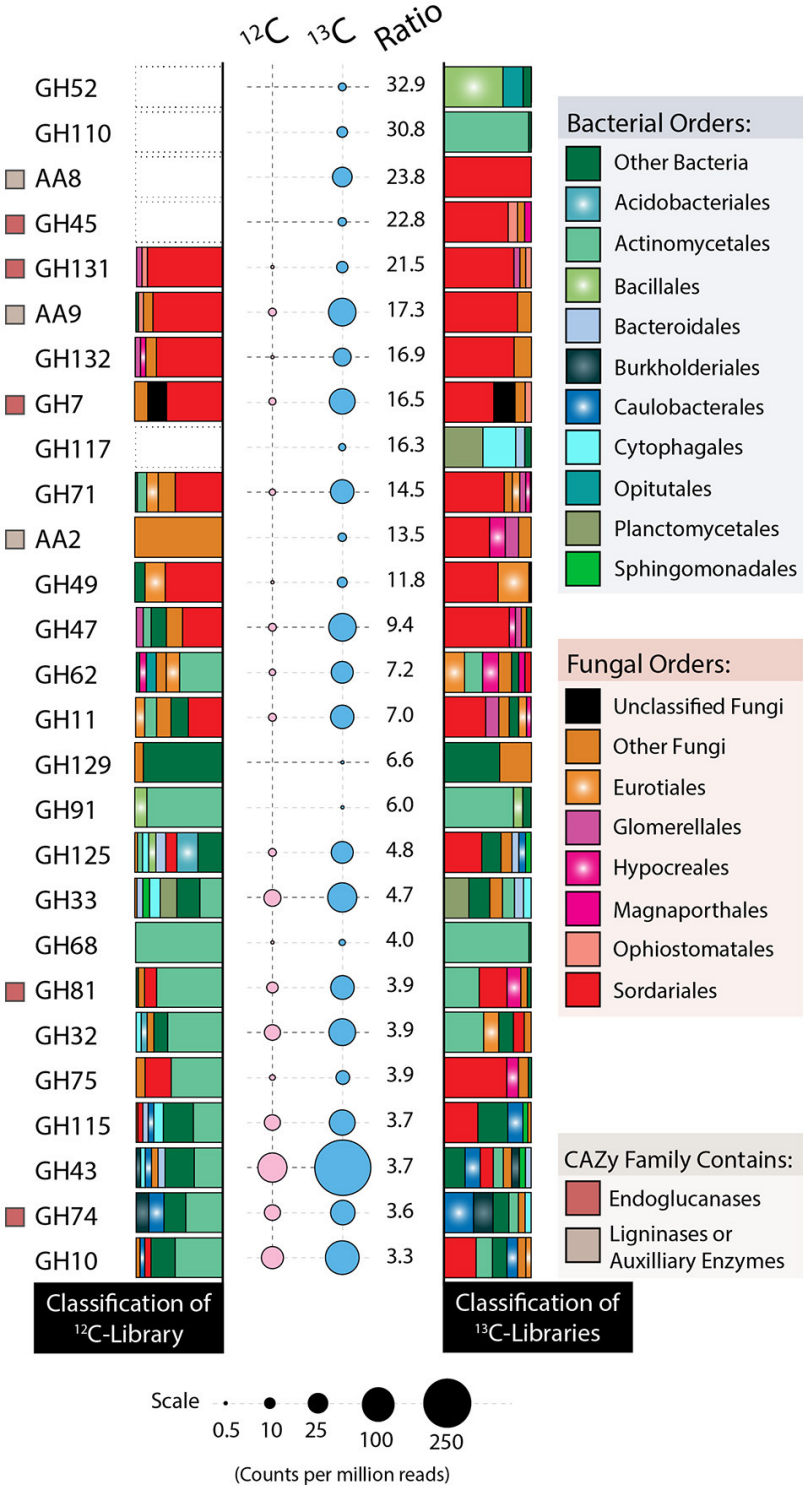
**Taxonomic affiliations of CAZy genes enriched in ^**13**^C- (blue) vs. ^**12**^C-control (pink) metagenomes**. Bubble area is scaled to counts per million among quality-filtered, unassembled reads, and the ratio corresponds to the relative counts between ^13^C and ^12^C metagenomes. CAZy gene families without a bubble had fewer than 0.5 counts per million reads. A beige square denotes lignin-modifying activity, while a red square denotes endoglucanase activity, based on www.cazy.org. Taxa comprising fewer than 5% of reads for any given family were binned as either “Other Bacteria” or “Other Fungi.”

Relative to the ^12^C-metagenome, unassembled ^13^C-metagenomes encoded double the number of glycosyl hydrolases (GH) and three-fold more GH families with reported endoglucanase activity. Five endoglucanase-containing families and lytic polysaccharide monooxygenases (AA9) were among the most enriched CAZy gene families in ^13^C-metagenomes (Figure 8). Lignin modifying enzymes, peroxidases (AA2) and iron reductase domains (AA8), were also highly enriched in ^13^C-metagenomes and were classified to the fungal order Sordariales, which contains the family Chaetomiaceae. The majority of differentially abundant GH genes were actinobacterial and fungal (Sordariales), while a lesser number were from Bacillales, Bacteroidales, Burkholderiales, Cytophagales, Opitutales, and Planctomycetes. Improved assemblies enabled the recovery of 10 taxonomically uniform MAGs of putatively cellulolytic bacteria (Figure 4; Table S3). The most complete were related to *Myceliophthora thermophila* (Ascomycota), *Kitasatospora* (Actinobacteria), Opitutaceae (Verrucomicrobia), *Herbaspirillum* (Betaproteobacteria), *Chthoniobacter* (Verrucomicrobia) and Caulobacteraceae (Alphaproteobacteria), though all likely represent, to an extent, a mixture of sub-populations.

## Discussion

### Effects of changes in community composition on cellulolytic activity

Our results, based on multiple data types, demonstrate that timber harvesting can effect long-term changes in the composition of cellulolytic populations that reduce the rate of cellulose decomposition. This finding builds upon previous research that showed long-term impacts of harvesting on the composition and putative lignocellulolytic capacity of soil decomposers (Cardenas et al., 2015; Leung et al., 2016) by directly linking changes in composition with functional activity. Our conclusions differ from the large-scale meta-analysis of harvesting impacts on microbial activity by Holdena and Treseder (2013), since we did not observe a correlation between changes in activity and microbial biomass. This difference is not contradictory, rather, our results reveal the relative influence of community composition on activity when broad changes in the structure of decomposer populations occur. The most pronounced change in cellulose-degrading populations was an increase in the relative abundance of saprotrophic fungi, namely Chaetomiaceae, and a decrease in bacteria, notably Gram-positive Streptomycetaceae. These trends were robust across all datasets from both microcosm experiments and field samples and, though an effect on these specific groups has not been previously identified, similar trends in the relative abundance of saprotrophic fungi and Actinobacteria have been observed in soil from harvested forests after seven (Lewandowski et al., 2015), 15 (Hartmann et al., 2009, 2012) and fourty years (Chatterjee et al., 2008). In one case, populations of Sordariales, including the family Chaetomiaceae, were increased in logged forests in Southeast Asian tropical forests (McGuire et al., 2015). These communities were similarly impacted by forest fire (Xiang et al., 2014) and large-scale tree die back due to insect herbivory (Štursová et al., 2014), suggesting these trends may be broadly associated with forest disturbance.

Our observation that lower cellulolytic activity corresponded with increased relative abundance of fungi is at odds with the conventional view that fungi are the most effective decomposers of recalcitrant plant polymers in soil (Štursová et al., 2012). There are several cases in which soil properties, nutrient availability and litter quality influenced whether decomposition was predominantly fungal or bacterial (Jastrow et al., 2007; Güsewell and Gessner, 2009; Strickland and Rousk, 2010). Consistent with our results, Strickland et al. (2009) found that the relative abundance of Sordariomycetes was negatively correlated with net respiration during litter decomposition, while the reverse was true for Actinobacteria. A decrease in respiration activity could result from lower inherent activity of certain members of Sordariomycetes or, that their manner of decomposition affects the overall utilization of cellulose by other populations by either changing the quality of OM or producing inhibitory by-products. Whatever may be driving this ecological phenomenon, it is certainly of interest for understanding the effects of forest disturbance on nutrient cycling, as well as how community composition affects the carbon sequestration in soils.

### Effects of harvesting on community composition

The greatest impacts of harvesting on cellulolytic populations (in microcosms and *in situ*) were observed at the highest intensity of OM removal (OM3), while intermediate levels (OM1 and OM2) were generally indistinguishable from each other. These observations agree with the conclusions from previous studies characterizing the effects of OM removal on soil communities (Hartmann et al., 2012; Cardenas et al., 2015; Leung et al., 2016). The general lack of difference between OM1 and OM2, which differ by the retention of woody debris in OM1, suggests that the changes we observed most likely result from long-term exposure to stress-inducing environmental conditions, like heat and dryness, which were most pronounced in OM3. Five years following harvesting at the Californian sites, soil temperatures in harvested plots were between 5% (OM1) and 40% (OM3) higher than in unharvested plots, and soil moisture declined throughout the soil column in OM3 to a level deemed an erosion hazard (Paz, 2001). In general, near-surface soils, like those sampled in this study, experience significant abiotic changes in the interim between harvest and canopy closure, including higher average and maximum temperatures and lower moisture content, as well as greater fluctuations (Childs and Flint, 1987; Adams et al., 1991; Kranabetter and Chapman, 1999; Redding et al., 2003; Kulmala et al., 2014). The overall increase in stress-tolerant taxa in harvested soils was indicative of the influence of long-term changes in soil temperature and moisture regimes.

The expansion of cellulolytic members of Chaetomiaceae (dark-septate, thermophilic fungi), exemplified the general shift in populations adapted to harsher conditions. Chaetomiaceae are prevalent in hot, arid environments (Powell et al., 2012) and fire-prone forests (Rajulu et al., 2014) with several characterized thermophilic species, such as *Myceliophthora thermophila* (Berka et al., 2011). Similarly, the dramatic increase in harvested plots of non-cellulolytic taxa which possess remarkable stress-tolerance, such as *Methylobacterium* (Nogueira et al., 1998; Rokitko et al., 2003) and *Geodermatophilus* (Sghaier et al., 2016), is consistent with the influence of harsher environmental conditions. The decline in the abundance of cellulolytic Verrucomicrobia and *Caulobacter* in harvested plots may also be explained by drier soils in the decades post-harvesting. Verrucomicrobial populations have been positively correlated with soil moisture (Buckley and Schmidt, 2001), and *Caulobacter* species are generally known as aquatic organisms and have been found to respond rapidly to soil wetting (Fazi et al., 2008). The consistency of these trends across multiple datasets within our study and with previously published data from LTSP installations in British Columbia indicates the long-term selection pressures for stress-tolerant groups present post-harvesting.

For the most part, the retention of woody debris (OM1 relative to OM2) did not cause major differences in cellulolytic activity or composition of cellulolytic populations. An increase in the diversity of wood rot fungi can occur after harvesting when woody debris is retained (Brazee et al., 2014), but a similar trend was not supported by our data. One major point of difference between OM1 and OM2 was the relative abundance of fungi and bacteria, where OM1 and REF both had significantly more bacterial biomass than OM2 and OM3. There was some evidence to suggest differences in organic matter factored since both REF and OM1 had higher total carbon content as well as a slightly higher C:N ratio. Yet, these differences, for the most part, were not statistically significant and the correlations of major bacterial and fungal taxonomic groups (Actinobacteria and Sordariomycetes) to C:N (−/−, respectively) and total carbon (±) were at odds with the trends in fungal and bacterial biomass among treatments. While our study was not designed to discern between the effects of OM retention on environmental conditions vs. on OM quality and quantity, the clearest impact of OM retention in our results was to mitigate changes in the relative abundance of stress-tolerant taxa.

Changes in cellulolytic populations differed in several ways from previous observations of the long-term effects of harvesting on whole soil communities. Firstly, a decrease in the ratio of Basidiomycota to Ascomycota is a common characteristic of soil fungal communities in the years and decades following timber harvesting (Bader et al., 1995; Hartmann et al., 2012; Štursová et al., 2014; McGuire et al., 2015) and following forest fire (Holdenb et al., 2013; Buscardo et al., 2015). Accordingly, we found a significant decrease in the ratio in harvested plots from field samples (i.e., *in situ*), but not in cellulolytic populations (i.e., ^13^C-pyrotag libraries). The lack of difference among cellulolytic populations may simply reflect the predominance of Ascomycota as cellulose degraders, which overshadowed any negative impacts of harvesting on cellulolytic Basidiomycota. In general, the predominance of cellulolytic Ascomycota in forest soils supports the hypothesis that the generally observed shift in the ratio of Basidiomycota and Ascomycota reflects a post-harvesting shift to a saprotroph-dominated system (Hartmann et al., 2012). Notably, the relative abundance of one of the two cellulolytic groups of Basidiomycota identified, *Clitopilus spp*., increased in OM3, suggesting some Basidiomycota may thrive in post-harvest conditions. Secondly, the diversity of cellulolytic populations was not impacted by harvesting, contrary to a similar study of the effects of prescribed burning which found a significant decrease in the diversity of cellulolytic taxa (Bastias et al., 2009). In general, soil microbial diversity does not differ between primary to secondary forests (Lauber et al., 2008; Paula et al., 2014; McGuire et al., 2015; Oliver et al., 2015), but is commonly reduced by the conversion of forest to agricultural land (Rodrigues et al., 2013). Thus, our findings for cellulolytic populations were in broad agreement with previous characterizations, suggesting diversity is less indicative of harvesting effects compared to shifts in community structure, in our case, toward stress-tolerant taxa.

### Composition of forest soil cellulolytic populations

The majority of taxa identified by SIP (~75%) had previously documented cellulolytic activity, including well-characterized groups, such as Actinobacteria, Bacteroidetes, Cytophaga, Myxococcales, and Sordariomycetes. Though the potential of non-cellulolytic taxa incorporating sufficient ^13^C-label via cross-feeding to be designated as cellulolytic cannot be ruled out in SIP experiments, this level of agreement with culture-dependent characterizations supports the effectiveness of SIP as a culture-independent method to link sequence data with function. The remaining taxa, not previously known to degrade cellulose, were largely associated with mineral layer soil, an atypical sample source for studies of cellulose-degradation. For example, well-characterized cellulolytic taxa, like Cytophaga and Actinobacteria, were associated with organic layer soils, while mineral layer-associated cellulolytic taxa belonged to candidate division FBP (Armatimonadetes), with no representative genome or isolate, and members of the ubiquitous, yet poorly characterized phylum, candidatus Saccharibacter (formerly TM7), of which we recovered a partial MAG (~0.4 Mb). Phyla with relatively few cultured representatives, such as Armatimonadetes, Verrucomicrobia, and Planctomycetes, were also mineral layer-associated, each possessing at least one representative characterized to degrade cellulose (Sangwan et al., 2004; Dedysh and Kulichevskaya, 2013; Lee et al., 2014). In terms of cellulolytic activity, microbial biomass in mineral soils exhibited higher activity per unit total biomass than organic layer soil, and, in mineral layer soil, ^13^C was primarily assimilated by bacteria. Thus, this study revealed mineral soil to possess active cellulolytic populations, distinct from those in the organic layer soils, comprised of poorly characterized cellulolytic taxa.

SIP-based designations of cellulolytic fungi also matched previously characterized cellulolytic taxa, such as *Humicola, Clitopilus*, and members of Chaetomiaceae. The putative assignment of a member of Sebacinaceae was novel, given members of this family are better known for their ectomycorrhizal associations. Yet, a few saprobic species of Sebacinaceae have been described, though they have not previously been reported to be cellulolytic (Oberwinkler et al., 2013; Weiß et al., 2016). The 46-Mb fungal MAG was the first eukaryotic genome of its size recovered from shotgun metagenomic data and, a size that resembles that of a *Myceliophthora thermophila* draft genome (~38.7 Mb; Berka et al., 2011). Our MAG encodes a number of CAZy families with endoglucanases, lytic polysaccharide monooxygenases (AA9), iron reductase domains (AA8) and peroxidases (AA2), which suggest a role in both cellulose and lignin decomposition that mirrors cultured representatives of *M. thermophila*, which can completely degrade lignocellulose and encode a diverse array of thermostable CAZymes (Babot et al., 2011; Berka et al., 2011). The recovery of multiple MAGs, including such a large one, demonstrates the power of combining SIP and shotgun metagenomics to investigate the function of taxa in highly diverse soil communities where metagenome assembly is otherwise typically poor.

## Conclusions

This study builds upon research that demonstrates consistent long-term impacts of harvesting on forest soil microbial communities. We provide evidence that OM retention during harvesting has the potential to minimize changes in soil cellulolytic populations by mitigating changes in environmental stressors such as heat and surface soil drying. The changes we observed were present one-year after canopy closure, coinciding with the end of the period when soils experienced the greatest exposure to abiotic changes. As changes in cellulolytic populations were ostensibly driven by abiotic factors, comparisons with the effects of other canopy-removing forms of natural disturbance, like wildfire, will be valuable in establishing perspectives on longer-term impacts. These perspectives are valuable to forestry management practices which are increasingly aligned with principles of emulating natural disturbance. The legacy of the microbial populations which flourish during the first two decades of forest regeneration, which we identify here, remain unknown. In the case of cellulolytic taxa, the effect could be strong as the early colonizers of decaying litter can influence succession and the quality of decomposition (Song et al., 2015). Further study of these changes may yield novel insights into the relative activity of fungi and bacteria in forest soils, given our unusual observation of decreased rates of cellulolytic activity with increased fungal participation. Ultimately, the impact of these groups will depend on their sustained activity as forests mature and their possible persistence across multiple harvests. Of likely equal importance is the time-frame for the repopulation of the taxa we found in decline. It is too early to tell whether the phenomena we describe have broader ecosystem effects that impact forest regeneration. Certainly, the populations identified by this study are candidates for future monitoring efforts and long-term research. And, given the consistency of our findings in California with previous LTSP research in British Columbia (Hartmann et al., 2012), our conclusions have broad implications for the long-term impacts of timber harvesting and may shape principles of forest stewardship.

## Author contributions

RW designed all experiments; collected all data; and performed all analysis and writing, except were stated otherwise. EC performed the binning of shotgun metagenomes. HL assisted in developing SIP-PLFA and SIP-DNA approaches. AS performed mass spectroscopy to quantify ^13^C content of DNA. LJ conducted respiration experiments. WM provided critical assistance in experimental design and in writing.

## Funding

Funding was provided by Genome Canada and Genome British Columbia. Support for RW was provided by an NSERC graduate scholarship, and for EC by a postdoctoral fellowship from the Tula Foundation.

### Conflict of interest statement

The authors declare that the research was conducted in the absence of any commercial or financial relationships that could be construed as a potential conflict of interest.
