# Corrigendum: Long-Term Enrichment of Stress-Tolerant Cellulolytic Soil Populations following Timber Harvesting Evidenced by Multi-Omic Stable Isotope Probing

**DOI:** 10.3389/fmicb.2017.01170

**Published:** 2017-06-20

**Authors:** Roland C. Wilhelm, Erick Cardenas, Hilary Leung, András Szeitz, Lionel D. Jensen, William W. Mohn

**Affiliations:** ^1^Department of Microbiology and Immunology, Life Sciences Institute, University of British ColumbiaVancouver, BC, Canada; ^2^Pharmaceutical Analytical Suite, Faculty of Pharmaceutical Sciences, University of British ColumbiaVancouver, BC, Canada

**Keywords:** timber harvesting, stable isotope probing, metagenomics, cellulose, decomposition, retention harvesting, disturbance ecology

The error occurred in the Materials and Methods section where we incorrectly reported a higher addition of ^13^C-labeled substrate than was added. The error does not affect any of our conclusions, but we felt it could mislead future researchers interested in utilizing SIP techniques. We corrected the error in two places (page 4 sections “Soil Respiration Assays” and “SIP Microcosms”) from “(10% w/w)” to “(1% w/w).” We utilized 10 mg ^13^C-cellulose per gram dry weight soil and inadvertently miscalculated the % w/w. The authors apologize for these mistakes and state that this does not change the scientific conclusions or merit of the article in any way.

## Conflict of interest statement

The authors declare that the research was conducted in the absence of any commercial or financial relationships that could be construed as a potential conflict of interest.

