# Supplementary material for: Long-Term Enrichment of Stress-Tolerant Cellulolytic Soil Populations following Timber Harvesting Evidenced by Multi-Omic Stable Isotope Probing
Source: Front Microbiol. 2017 Apr 11;8:537. doi: 10.3389/fmicb.2017.00537 (PMC5386986; doi:10.3389/fmicb.2017.00537)
Supplement: Supplementary file 1 [file Table1.PDF]

Table S1. Environmental and soil characteristics of the three Californian sampling sites and microbial activity of corresponding soils. Values denoted by letters are statistically significant ($p < 0.05$) based on Tukey's Honest Significant Difference.

		Blodgett	Brandy City	Lowell Hill
Site Data	Year Established	1994	1995	1995
	Lat / Long	38.88N / 120.64W	39.55N / 121.04W	39.26N / 120.78W
	Elevation	1350 m	1135 m	1268 m
	Soil type	Mesic Ultic Haploxeralfs (Loamy)		
Soil composition (n=9)	Average Percent Carbon	$8^{A} \pm 0.36$	$7^{B} \pm 0.15$	$4.7^{C} \pm 0.14$
	Average Percent Nitrogen	$0.37^{A} \pm 0.019$	$0.32^{B} \pm 0.007$	$0.2^{C} \pm 0.004$
	Average C:N Ratio	22.1 ± 0.45	22.2 ± 0.42	23.5 ± 0.77
	Average pH	5.69 ± 0.04	5.77 ± 0.08	5.81 ± 0.09
Respiration (n=36)	Average mg CO2 per g soil	$1.13^{A} \pm 0.05$	1.02 ± 0.04	$0.97^{B} \pm 0.05$
PLFA Biomass (n = 12)	Average Delta ^{13}C	$1400^{A} \pm 100$	$3300^{B} \pm 400$	$2500^{B} \pm 300$
	Total ^{13}C Biomass (µmol ^{13}C per g soil)	0.33^{A}	0.63^{B}	0.53
	Total ^{12}C Biomass (µmol ^{12}C per g soil)	46.5^{A}	25.8^{B}	21.3^{B}

Technical error (S.D.) for Delta-values is 80, which is equivalent to an average of ± 0.0003 µmols C
