# Supplementary material for: Long-Term Enrichment of Stress-Tolerant Cellulolytic Soil Populations following Timber Harvesting Evidenced by Multi-Omic Stable Isotope Probing
Source: Front Microbiol. 2017 Apr 11;8:537. doi: 10.3389/fmicb.2017.00537 (PMC5386986; doi:10.3389/fmicb.2017.00537)
Supplement: Supplementary file 2 [file Table2.PDF]

Table S2. Overview of metagenomic data from ^{12}C-library ('Control') and ^{13}C-libraries ('OM1','OM3' and 'REF'). Percent assembly represents the percentage of reads mapping back to assembled data, i.e. the percent of reads used in the assembly from each metagenome.

	Control	REF	OM1	OM3
MG-RAST Acession	4564580.3	4564582.3	4565365.3	4564581.3
European Nucleotide Archive Sample Accession	ERS1099581	ERS1099584	ERS1099582	ERS1099583
Total Number of Quality Filtered Reads (x 10^6)	60.5	88.9	45.9	90.8
Total Contigs > 500nt	268	35,758	15,380	26,616
Percent Assembly	0.93%	28.9%	22.6%	17.0%
Alpha-diversity	405	325	295	383
