# Supplementary material for: Long-Term Enrichment of Stress-Tolerant Cellulolytic Soil Populations following Timber Harvesting Evidenced by Multi-Omic Stable Isotope Probing
Source: Front Microbiol. 2017 Apr 11;8:537. doi: 10.3389/fmicb.2017.00537 (PMC5386986; doi:10.3389/fmicb.2017.00537)
Supplement: Supplementary file 3 [file Table3.PDF]

Table S3. Details of draft genome bins recovered from metagenomics assemblies. 'Completeness' is a measure of the total number of house-keeping genes present from a list of single copy 'essential' genes (Albertsen *et al.* 2013). 'Redundancy' refers to the number of times those house-keeping genes recurred. The final four columns correspond to the percentage of reads mapped from the respective metagenomic samples to each genome bin.

Taxonomic Affiliation of Draft Genome Bin	MEGAN Classification (% of bases assigned)	Scaffold Accession Numbers (at ENA)	Size of Partial Genome (Mb)	Number of Contigs	Largest Scaffold (Kb)	Completeness (%)	Redundancy (%)	Control (%)	OM1 (%)	OM3 (%)	Reference (%)
Myceliophthora thermophila	Ascomycota (99%)	FJWA01000001-FJWA01010187	46.2	10187	32.1	100	24	0.47	14.07	9.67	7.97
Kitasatospora sp.	Actinobacteria (79%)	FJVZ01000001-FJVZ01001553	8.1	1553	31.7	100	21	0.08	0.54	0.28	6.05
Opitutaceae spp.	Verrucomicrobia (78%)	FJVV01000001-FJVV01001035	5.7	1035	46.9	100	18	0.00	0.03	0.03	1.71
Herbaspirillum sp.	β - Proteobacteria (100%)	FJVX01000001-FJVX01001352	4.3	1352	19.3	89	24	0.00	1.73	0.02	0.06
Chthoniobacter sp. 1	Verrucomicrobia (74%)	FJVU01000001-FJVU01000901	4.0	901	21.9	66	24	0.00	0.09	0.05	1.11
Caulobacteraceae spp.	α - Proteobacteria (96%)	FJVW01000001-FJVW01001105	3.3	1105	14.8	76	19	0.00	0.06	0.04	0.72
Heterogeneous Bin	Cand. Saccharibacteria (27%)	FJVY01000001-FJVY01000803	2.4	103	37.7	94	32	0.01	0.27	0.18	0.64
Arthrobacter sp.	Actinobacteria (60%)	FJVT01000001-FJVT01000548	0.8	548	5.3	10	0	0.01	0.07	0.15	0.06
Oxalobacteraceae spp.	β - Proteobacteria (96%)	FJVS01000001-FJVS01000283	0.4	283	3.4	0	NA	0.00	0.03	0.01	0.08
Candidatus Saccharibacteria	Cand. Saccharibacteria (56%)	FJVR01000001-FJVR01000103	0.4	803	16.0	38	34	0.00	0.01	0.02	0.15
Chthoniobacter sp. 2	Verrucomicrobia (65%)	NA	0.4	119	6.0	5	0	0.00	0.01	0.00	0.11
