# Supplementary material for: Long-Term Enrichment of Stress-Tolerant Cellulolytic Soil Populations following Timber Harvesting Evidenced by Multi-Omic Stable Isotope Probing
Source: Front Microbiol. 2017 Apr 11;8:537. doi: 10.3389/fmicb.2017.00537 (PMC5386986; doi:10.3389/fmicb.2017.00537)
Supplement: Supplementary file 4 [file DataSheet1.PDF]

Iso*Life*

Iso*Life* bv, P.O. Box 349 NL-6700 AH Wageningen

Visiting address: Droevendaalsesteeg 1, Bldg 107, NL-6708 PB Wageningen, The Netherlands

Info@isolife.nl   Tel: +31 (0) 317 480512   www.isolife.eu

CERTIFICATE OF ANALYSIS

Article name:	**U-^{13}C Cellulose high DP**
Origin:	Maize stem *(Zea mays L.)*
Catalogue number:	U-10508
Lot number:	0901–0273
Isotopic enrichment:	Uniform, 97 atom % ^{13}C
Chemical purity:	58 % glucose (w/w)
DP (average; by viscosity in cueen):	2,100
Average Mol. Wt.	350 kD
Crystallinity (by WAXS):	optional
Carbohydrate analysis:	optional
Residual lignin:	4.4 % (w/w)
Appearance:	fluffy, fibrous, off-white

April 15, 2010

Iso*Life*

Chamber of Commerce Arnhem Trade Register no 09147374
