# Supplementary material for: Long-Term Enrichment of Stress-Tolerant Cellulolytic Soil Populations following Timber Harvesting Evidenced by Multi-Omic Stable Isotope Probing
Source: Front Microbiol. 2017 Apr 11;8:537. doi: 10.3389/fmicb.2017.00537 (PMC5386986; doi:10.3389/fmicb.2017.00537)
Supplement: Supplementary file 8 [file DataSheet5.DOCX]

*Preparation of ^13^C-labeled Cellulose*

Cellulose substrates were produced by feeding *Gluconacetobacter xylinus* str. KCCM 10100 with ^13^C-labeled glucose (99 atom % ^13^C, Cambridge Isotope Laboratories, MA, USA) and unlabeled glucose in Yamanaka media under conditions outlined in Ruka *et al.* (2012). Cellulose was purified according to Pinnel *et al.*, (2011) with an additional repetition of boiling in 1% sodium hydroxide and an increase to 4 hrs of boiling time. Cellulose from maize (> 97 atom % ^13^C *Zea mays*, U-10508, Lot #: 0901–0273) was initially sourced from IsoLife (Wageningen, The Netherlands), but was abandoned due to substantial impurity, namely it was only 58% glucose (w/w) (see Supplementary Data 1). Bacterial cellulose has similar mechanical properties to plant cell walls, comparable polymer length (3000 – 9000 units) and crystallinity (80-90% crystalline) (Chanliaud *et al.* 2002) and was previously used in SIP applications (El Zahar Haichar *et al.* 2007; Pinnel *et al.* 2014).

*Stable Isotope Probing Phospholipid Fatty Acids*

PLFAs were extracted from 0.75 g (organic) or 1.0 g (mineral) dry wt soil according to Bligh and Dyer (1959) and the ^13^C-content was analyzed using IRMS (UBC Stable Isotope Facility) ported with gas chromatography as detailed in Churchland et al. (2013) with the following exceptions: (i) methyl undecanoate (c11:0) was used for the internal standard, (ii) quantitation was based on an average of three serial dilutions of undecanoate, nonadecanoate (c19:0), and methyl cis-13-docosenoate (c22:1ω9). Peak identification was based on retention time compared against two reference standards: bacterial acid methyl-ester standard (47080-0; Sigma–Aldrich, St. Louis) and a 37-Component fatty acid methyl-ester mix (47885-U; Sigma–Aldrich, St. Louis). Unidentifiable enriched peaks, termed “unidentified fatty acids” (UFA), were included in analysis if they met the following conditions: i) detection in > 3 samples, ii) average δ ^13^C > +50 ‰ and iii) confirmed as long-chain alkane methyl esters by GC-MS using the identical instrumentation and method as in Leckie *et al.,* (2004). Taxonomic affiliations of specific PLFAs were assigned according to Högberg *et al.,* (2013), with c18:1ω9 and c18:3ω6 added as fungal markers according to Ruess and Chamberlain (2010). All comparisons between taxonomic groups were performed using values normalized to total PLFA content. All SIP-PLFA raw data was processed identically in R using scripts that can be found at: https://github.com/roli-wilhelm.

*Stable Isotope Probing, DNA Pyrosequencing and Metagenomic Library Preparation*

DNA was extracted from 0.5 g soil with the manufacturer’s recommended protocol for the FastDNA™ Spin Kit for Soil (MPBio, Santa Ana, CA). The mass and the atom % ^13^C of DNA extracts were measured with UHPLC-MS-MS according to Wilhelm *et al.* (2014). DNA extracts from replicates within each site were pooled in equal concentrations and unlabeled controls were identically processed (n = 24 x 2). ^13^C-enriched DNA was recovered by density gradient ultracentrifugation according to methods outlined in Neufeld *et al.* (2007). Full details of equipment and methods can be found in Wilhelm *et al.* (2014). Important modifications include: i) the amount of DNA applied to the column was standardized according to the degree of ^13^C enrichment, resulting in between 6 – 10 µg of DNA used per sample and ii) the resuspension of DNA from the cesium chloride solution was performed more efficiently and effectively using Amicon Ultra-0.5 mL filters (EMD Millipore, MA, USA) in place of PEG_6000_. Heavy fractions (1.727-1.735 g/mL, typically fractions 1-7) were pooled (max 500 µL) and concentrated in the filter columns by centrifugation for 3 min at 14,000 rcf. Samples were then washed three times with PCR-grade water. Pyrotag libraries from all 48 samples were prepared by amplification of the bacterial small-subunit ribosomal RNA gene (16S; V1-V3) as well as the fungal internal transcribed spacer region (ITS2), as described by Hartmann et al. (2012). The 454-pyrosequencing was performed at Génome Québec. Control samples from incubations without ^13^C-cellulose were treated identically, however insufficient DNA was recovered from heavy fractions for PCR, resulting in the need to pool additional fractions (~1-9). The efficacy of separating enriched DNA from high-GC bacteria was tested by spiking in DNA from *Nocardioides sp.* (70% GC; IMG Taxon ID: 2519899648) at 5, 10, 15 and 20% (v/v) of DNA applied to the gradient. After purification, the ^13^C-enriched DNA yielded fewer than 1% pyrotags corresponding to the isolates, though the number of sequences from isolates correlated with the proportion of DNA added. Metagenomic libraries were prepared from 40-50 ng of enriched DNA using the Nextera DNA Sample Preparation Kit (Illumina Inc., CA, USA). Four shotgun metagenome libraries were constructed from mineral layer soil by pooling all replicates and sites for treatments: REF, OM1, OM3 and unlabeled REF sites. These libraries were multiplexed on two lanes of Illumina HiSeq (2 x 100-bp), yielding 285 million paired-end reads at UBCSeq. There was insufficient DNA to create metagenomic libraries from organic soil layer samples. As part of a larger project, pyrotag and shotgun metagenome libraries were generated from the original soil samples (i.e. not incubated). All raw pyrotag sequence files were uploaded to the European Sequencing Archive and are publically available using accession numbers, bacterial: ERS803692-ERS803739, and fungal: ERS803740-ERS803786. All metagenomic data is available from MG-RAST using accession numbers: 4564580.3 (Control), 4564581.3 (OM3), 4564582.3 (REF), 4565365.3 (OM1).

*Statistical and Bioinformatic Analysis*

Statistics were performed using R v. 3.1.0 (R Core Team, 2014) with general reliance on the capabilities provided in the following packages: reshape2, ggplot2, plyr (Wickam, 2007; Wickam, 2009; Wickam, 2011) and Hmisc (Harrell and Dupont, 2015). Prior to statistical testing, data was tested for normality and variance. Parametric analysis of variance tests from vegan were used to assess differences between treatments. P-values were adjusted according to the Benjamini & Hochberg (1995) false discovery rate (FDR) correction where necessary. Pyrotag libraries were quality filtered and processed according to the Schloss “454 SOP” (accessed January 2014; Schloss *et al.* 2011). To produce count tables, 16S rRNA gene libraries were clustered into operational taxonomic units (OTUs) at 0.01% dissimilarity. Due to the hypervariability of the ITS region, forming OTUs even at 0.05% dissimilarity resulted in very sparse count tables. Therefore, we binned ITS sequences based on taxonomic classification. OTUs were then filtered by abundance to remove any OTU with fewer than five counts across all 48 samples and OTUs that did not occur in more than three samples. Bacterial OTUs were assigned taxonomic classifications using Greengenes (database gg_13_5_99; May 2013), and fungal OTUs, using the mother-formatted release of UNITE (sh_mothur_release_08.12.2013; August 2013). Permutational multivariate analysis of variance (PERMANOVA; ‘adonis’ in Vegan; Oksanen et al. 2015) was performed on Bray-Curtis dissimilarities based on OTU tables. NMDS plots were prepared using Bray-Curtis dissimilarity with “metaMDS” (from Vegan) for both 16S rRNA gene and ITS libraries. We used three methods to identify OTUs differentially abundant between ^12^C-control and ^13^C-enriched library samples: differential gene expression analysis with mean-variance stabilization using the negative binomial distribution (Anders and Huber 2010: “DESeq” in R), differential gene expression analysis with mean-variance stabilization using precision weighting (Ritchie 2015: “limma-voom” in R) and uncorrected, averaged relative abundance. An OTU deemed enriched enrOTU had at least a 3-fold higher relative abundance in ^13^C versus ^12^C libraries. Phylogenetic libraries were managed using the R package “phyloseq” for ease of use (McMurdie and Holmes 2014). Indicator species analysis was performed using the R package “indicspecies” (Cáceres and Legendre 2009). Metagenomic data was quality filtered using FastX-Toolkit (v.0.7; http://hannonlab.cshl.edu/fastx-toolkit) and annotated with MG-RAST (Meyer *et al.* 2008). All records of carbohydrate-active enzymes in the CAZy database (Cantarel *et al.* 2008; <http://www.cazy.org/>) were downloaded, and protein sequences were retrieved from genbank with a custom script (on 2013-07-13). The CAZy database was queried using ‘BlastX’ for all metagenomics reads using DIAMOND (Buchfink *et al.* 2014). The following glycosyl hydrolase families were used when profiling for endoglucanase activity: GH5, 6, 7, 8, 9, 12, 26, 44, 45, 48, 51, 61, 74, 81 and 131. Metagenomic reads were classified using MEGAN (v. 5.10.1; Huson *et al.* 2007) using output from BlastX queries against the GenBank non-redundant protein database (downloaded 2014-10-13). Individual metagenomic libraries as well as a composite of all SIP-libraries (super assembly) were assembled using Ray-meta (Boisvert *et al.* 2012). Subsequent binning of contigs into putative draft genomes was performed with Metawatt (v. 2.1; Strous *et al.* 2012), based on tetranucleotide frequency, and MetaBAT (v. 0.18.6; Kang *et al.* 2014), by both tetranucleotide frequency and by covariance in read abundance mapped to the super assembly. Raw reads were then re-mapped onto draft genome bins using Bowtie2 (Langmead *et al.* 2012), and any mapped reads were then re-assembled separately. The re-assembled contigs were merged with draft bins (which contained contigs from the super assembly) using minimus2 from the AMOS package (Treangen *et al.* 2011) to improve the overall quality of the draft. Finally, each draft bin had both SIP and *in situ* metagenomic data mapped to it using Bowtie2 in order to calculate the relative abundance of these taxa across harvesting treatments. All R analyses and raw data can be found at:

<https://github.com/Roli-Wilhelm/Harvesting_Impacts_Cellulolytic_Community>

**References**

Anders S, Huber W. (2010) Differential expression analysis for sequence count data. *Genome Biol* **11:** R106.

Benjamini Y & Hochberg Y (1995) Controlling the false discovery rate: a practical and powerful approach to multiple testing. *J R Stat Soc B* **57***:* 289-300.

Bligh EG, Dyer WJ. (1959) A rapid method of total lipid extraction and purification. *Can J Biochemi Phys* **37**: 911-917.

Boisvert S, Raymond F, Godzaridis É, Laviolette F & Corbeil J (2012) Ray Meta: scalable de novo metagenome assembly and profiling. *Genome Biol* **13**: R122.

Buchfink B, Xie C & Huson DH (2015) Fast and sensitive protein alignment using DIAMOND. *Nat Meth* **12**: 59-60.

Cantarel BL, Coutinho PM, Rancurel C, Bernard T, Lombard V & Henrissat B (2009) The Carbohydrate-Active EnZymes database (CAZy): an expert resource for Glycogenomics. *Nucl Acids Res* **37**: D233-D238.

Chanliaud E, Burrows K, Jeronimidis G & Gidley M (2002) Mechanical properties of primary plant cell wall analogues. *Planta* **215**: 989-996.

Churchland C, Grayston SJ, Bengtson P. (2013) Spatial variability of soil fungal and bacterial abundance: consequences for carbon turnover along a transition from a forested to clear-cut site. *Soil Biol Biochem* **63**: 5-13.

De Caceres M, Legendre P. (2009). Associations between species and groups of sites: indices and statistical inference. Ecology, http://sites.google.com/site/miqueldecaceres/

Pinnel, L.J., Dunford, E.A., Ronan, P., Hausner, M. and Neufeld, J. (2014) Recovering glycoside hydrolase genes from active tundra cellulolytic bacteria. *Can J Microbiol*, **60**: 469-476.

El Zahar Haichar F, Achouak W, Christen R*, et al.* (2007) Identification of cellulolytic bacteria in soil by stable isotope probing. *Environ Microbiol* **9**: 625-634.

Harrell, F.E. and Dupont, C. (2015). Hmisc: Harrell Miscellaneous. R package version 3.17-1. https://CRAN.R-project.org/package=Hmisc

Hartmann M, Howes CG, VanInsberghe D, Yu H, Bachar D, Christen R *et al.* (2012) Significant and persistent impact of timber harvesting on soil microbial communities in Northern coniferous forests. *ISME J* **6**: 2199-2218.

Högberg M, Högbom L, Kleja D. (2013) Soil microbial community indices as predictors of soil solution chemistry and N leaching in Picea abies (L.) Karst. forests in S. Sweden. *Plant Soil* **372**: 507-522.

Huson DH, Auch AF, Qi J & Schuster SC (2007) MEGAN analysis of metagenomic data. *Genome Res* **17**: 377-386.

Kang DD, Froula J, Egan R & Wang Z (2014) A robust statistical framework for reconstructing genomes from metagenomic data. *bioRxiv* 011460.

Langmead B & Salzberg SL (2012) Fast gapped-read alignment with Bowtie 2. *Nat Meth* **9**: 357-359.

Leckie SE, Prescott CE, Grayston SJ, Neufeld JD & Mohn WW (2004) Comparison of chloroform fumigation-extraction, phospholipid fatty acid, and DNA methods to determine microbial biomass in forest humus. *Soil Biol Biochem* **36**: 529-532.

McMurdie PJ & Holmes S (2013) phyloseq: An R Package for Reproducible Interactive Analysis and Graphics of Microbiome Census Data. *PLoS ONE* **8**: e61217.

Meyer F, Paarmann D, D'Souza M*, et al.* (2008) The metagenomics RAST server–a public resource for the automatic phylogenetic and functional analysis of metagenomes. *BMC Bioinformatics* **9**: 386.

Neufeld JD, Vohra J, Dumont MG, Lueders T, Manefield M, Friedrich MW *et al.* (2007) DNA stable-isotope probing. *Nat Protoc* **2**: 860-866.

Oksanen J, Blanchet GF, Kindt R, Legendre P, Minchin PR, O'Hara RB *et al.* (2015). vegan: Community Ecology Package. R package version 2.3-2. https://CRAN.R-project.org/package=vegan

R Core Team. (2015). R: A language and environment for statistical computing. R Foundation for Statistical Computing, Vienna, Austria. https://www.R-project.org/

Ritchie ME, Phipson B, Wu D, Hu Y, Law CW, Shi W *et al.* (2015). limma powers differential expression analyses for RNA-sequencing and microarray studies. *Nucl Acids Res* **43**: e47.

Ruess L, Chamberlain PM. (2010) The fat that matters: Soil food web analysis using fatty acids and their carbon stable isotope signature. *Soil Biol Bioch* **42**: 1898-1910.

Ruka DR, Simon GP & Dean KM (2012) Altering the growth conditions of Gluconacetobacter xylinus to maximize the yield of bacterial cellulose. *Carbohyd Polym* **89**: 613-622.

Schloss PD, Westcott SL, Ryabin T, Hall JR, Hartmann M, Hollister EB *et al.* (2009) Introducing mothur: open-source, platform-independent, community-supported software for describing and comparing microbial communities. *Appl Environ Microbiol* **75**: 7537-7541.

Strous M, Kraft B, Bisdorf R & Tegetmeyer HE (2012) The binning of metagenomic contigs for microbial physiology of mixed cultures. *Front Microbiol* **3**.

Treangen TJ, Sommer DD, Angly FE, Koren S & Pop M (2011) Next generation sequence assembly with AMOS. *Curr Protoc Bioinformatics* 11.18. 11-11.18. 18.

Wilhelm R, Szeitz A, Klassen TL, Mohn WW. (2014) Sensitive, Efficient Quantitation of 13C-Enriched Nucleic Acids via Ultrahigh-Performance Liquid Chromatography–Tandem Mass Spectrometry for Applications in Stable Isotope Probing. *Appl Environ Microbiol* **80**: 7206-7211.

Wickham, H. (2007). Reshaping Data with the reshape Package. *J Stat Soft*, **21:** 1-20.

Wickham, H. (2011). The Split-Apply-Combine Strategy for Data Analysis. *J Stat Soft*, **40**: 1-29.

Wickham, H. (2009) ggplot2: Elegant Graphics for Data Analysis. Springer-Verlag New York, 2009.
