# Supplementary material for: Long-Term Enrichment of Stress-Tolerant Cellulolytic Soil Populations following Timber Harvesting Evidenced by Multi-Omic Stable Isotope Probing
Source: Front Microbiol. 2017 Apr 11;8:537. doi: 10.3389/fmicb.2017.00537 (PMC5386986; doi:10.3389/fmicb.2017.00537)

Figure S1. Photographs capturing the initial conditions following harvesting for each degree of OM removal (A-C) and the appearance of the forest when sampling was conducted (~15 years after reforestation) (D). Image A illustrates harvesting treatment ‘OM1’ as well as the efforts to minimize soil compaction. Image B and C illustrate harvesting treatment ‘OM2’ and ‘OM3’, respectively. [photo credit: Dr. Matt Busse; mbusse@fs.fed.us]

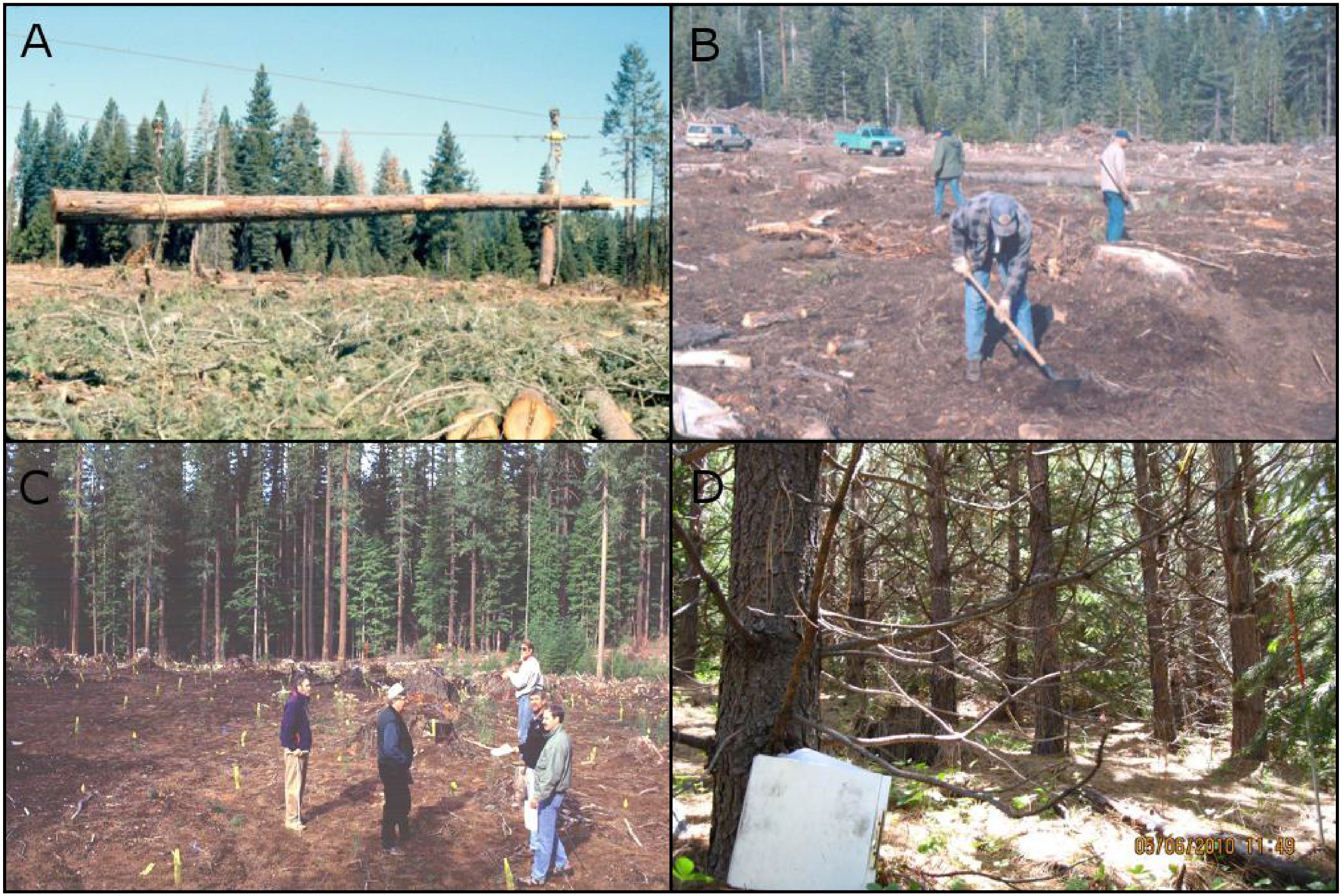

Supplement: Supplementary file 9 [file Image1.pdf]
