# Supplementary material for: Long-Term Enrichment of Stress-Tolerant Cellulolytic Soil Populations following Timber Harvesting Evidenced by Multi-Omic Stable Isotope Probing
Source: Front Microbiol. 2017 Apr 11;8:537. doi: 10.3389/fmicb.2017.00537 (PMC5386986; doi:10.3389/fmicb.2017.00537)

Figure S2. Comparisons of (A) ^{13}C-enrichment of soil DNA extract and (B) total DNA recovery from fraction F1-F7 between microcosms fed ^{12}C- and ^{13}C-cellulose. Consult Wilhelm *et al.* (2014) for additional comparisons and characterizations of successful separation and recovery of enriched nucleic acids.

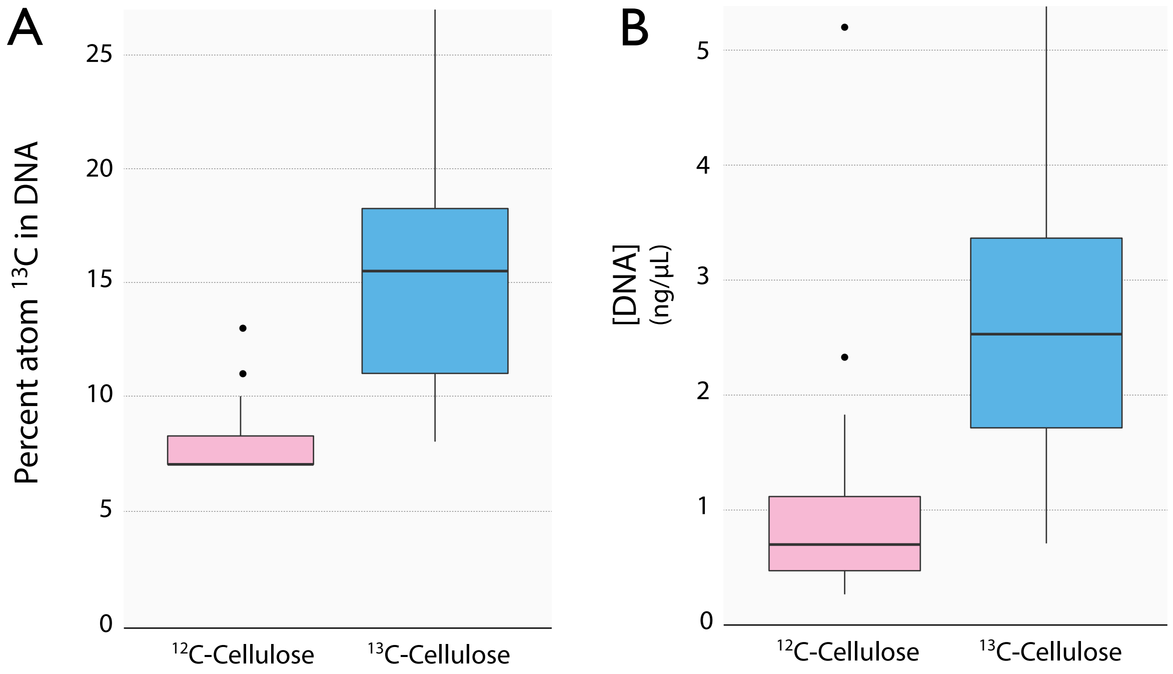

Supplement: Supplementary file 10 [file Image2.pdf]
