# Supplementary material for: Long-Term Enrichment of Stress-Tolerant Cellulolytic Soil Populations following Timber Harvesting Evidenced by Multi-Omic Stable Isotope Probing
Source: Front Microbiol. 2017 Apr 11;8:537. doi: 10.3389/fmicb.2017.00537 (PMC5386986; doi:10.3389/fmicb.2017.00537)

Figure S3. Plots of Shannon-Wiener diversity and Chao1 richness estimates for bacterial and fungal communities among harvesting treatments according soil layer for ^{13}C-, ^{12}C- and *in situ* libraries. The estimation for a given sample was based on an average of 500 calculations on OTU profiles rarefied to an equal sequencing depth. No statistically supported pairwise contrasts among treatments were found.

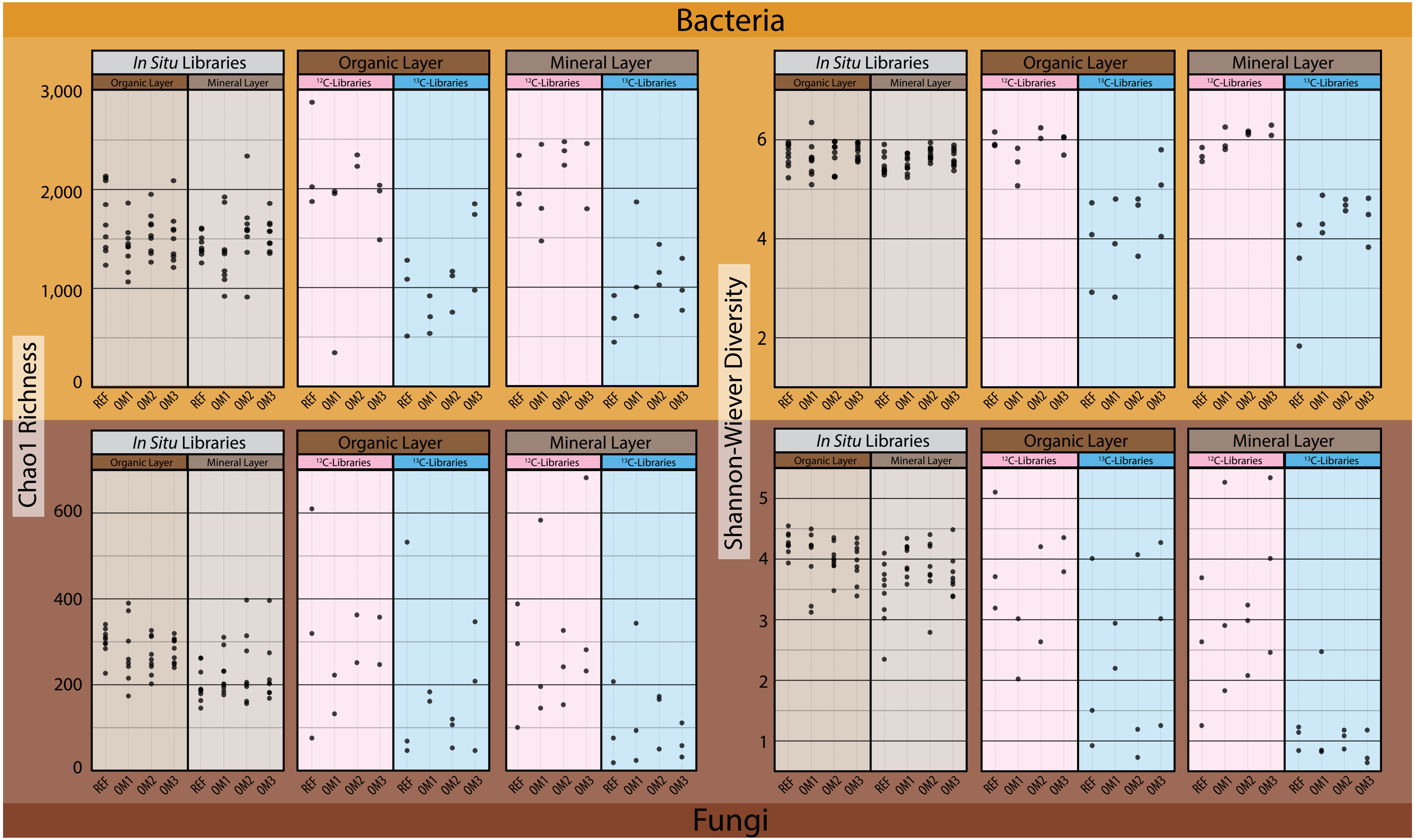

Supplement: Supplementary file 11 [file Image3.pdf]
