# Supplementary material for: Long-Term Enrichment of Stress-Tolerant Cellulolytic Soil Populations following Timber Harvesting Evidenced by Multi-Omic Stable Isotope Probing
Source: Front Microbiol. 2017 Apr 11;8:537. doi: 10.3389/fmicb.2017.00537 (PMC5386986; doi:10.3389/fmicb.2017.00537)
Supplement: Supplementary file 12 [file Image4.pdf]

**Figure S4**. Barplot of the percent variance ($R^2$) explained by each factor based on perMANOVA ($n_{perm}$ = 1000) using Bray-Curtis dissimilarities for either 16S rRNA gene or ITS pyrotag libraries. Only factors with statistical support ($p < 0.05$) factors were included in the plot. The full tabular perMANOVA results are included.

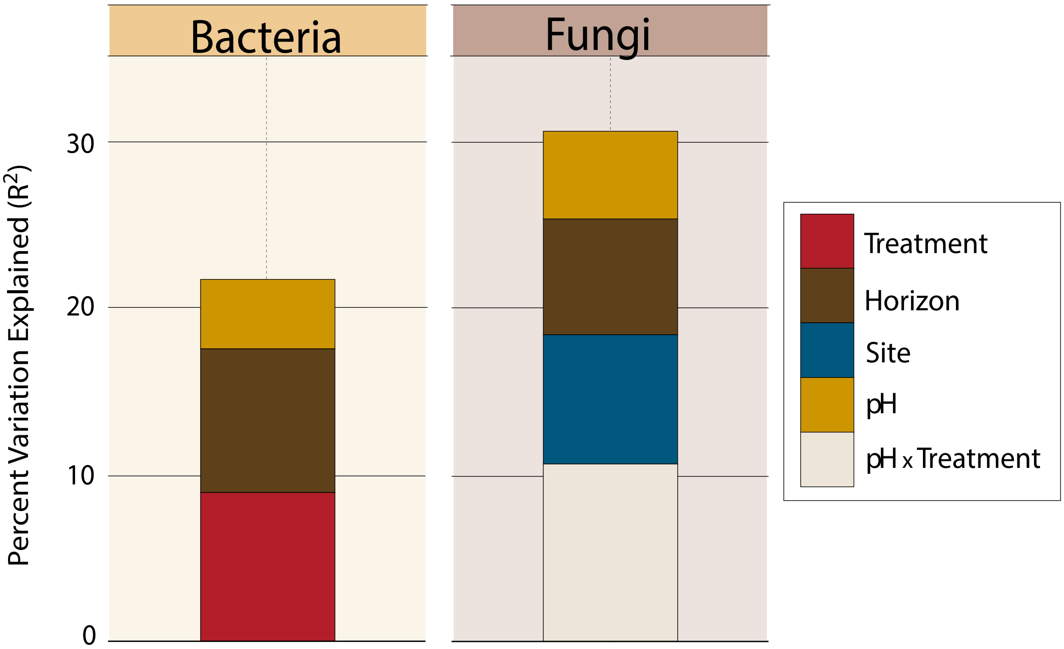


| Fungi | Df | Sum of Sqs | F.Model | $R^2$ | p-value |
|---|---|---|---|---|---|
| pH | 1 | 0.56 | 2.64 | 0.062 | 0.005 |
| Treatment | 3 | 0.92 | 1.43 | 0.102 | 0.091 |
| Horizon | 1 | 0.70 | 3.28 | 0.078 | 0.006 |
| C:N | 1 | 0.36 | 1.66 | 0.040 | 0.095 |
| Site | 2 | 0.77 | 1.80 | 0.086 | 0.041 |
| pH x Treatment | 3 | 0.99 | 1.54 | 0.110 | 0.053 |
| pH x Horizon | 1 | 0.19 | 0.90 | 0.021 | 0.500 |
| Treatment x Horizon | 2 | 0.61 | 1.42 | 0.068 | 0.130 |
| pH x C:N | 1 | 0.12 | 0.57 | 0.014 | 0.850 |
| Treatment x C:N | 3 | 0.35 | 0.81 | 0.039 | 0.68 |
| Residuals | 16 | 9.00 | - | 0.381 | - |

| Bacteria | Df | Sum of Sqs | F.Model | $R^2$ | p-value |
|---|---|---|---|---|---|
| pH | 1 | 0.63 | 1.90 | 0.046 | 0.002 |
| Treatment | 3 | 1.30 | 1.30 | 0.094 | 0.036 |
| Horizon | 1 | 1.24 | 3.74 | 0.090 | 0.001 |
| C:N | 1 | 0.36 | 1.09 | 0.026 | 0.301 |
| Site | 2 | 0.76 | 1.14 | 0.055 | 0.182 |
| pH x Treatment | 3 | 1.08 | 1.08 | 0.078 | 0.278 |
| pH x Horizon | 1 | 0.38 | 1.15 | 0.028 | 0.224 |
| Treatment x Horizon | 2 | 0.57 | 0.85 | 0.041 | 0.831 |
| pH x C:N | 1 | 0.29 | 0.88 | 0.021 | 0.666 |
| Treatment x C:N | 3 | 0.83 | 0.83 | 0.060 | 0.887 |
| Residuals | 19 | 6.32 | - | 0.459 | - |
