# Supplementary material for: Long-Term Enrichment of Stress-Tolerant Cellulolytic Soil Populations following Timber Harvesting Evidenced by Multi-Omic Stable Isotope Probing
Source: Front Microbiol. 2017 Apr 11;8:537. doi: 10.3389/fmicb.2017.00537 (PMC5386986; doi:10.3389/fmicb.2017.00537)

**Figure S5**. Relative abundances of taxa in LTSP sites in British Columbian from Hartmann *et al.* (2012) which correspond to indicator taxa identified in the present study. Data taken directly 'Supplementary Data 3' (Bacteria) and 'Supplementary Data 4' (Fungi).

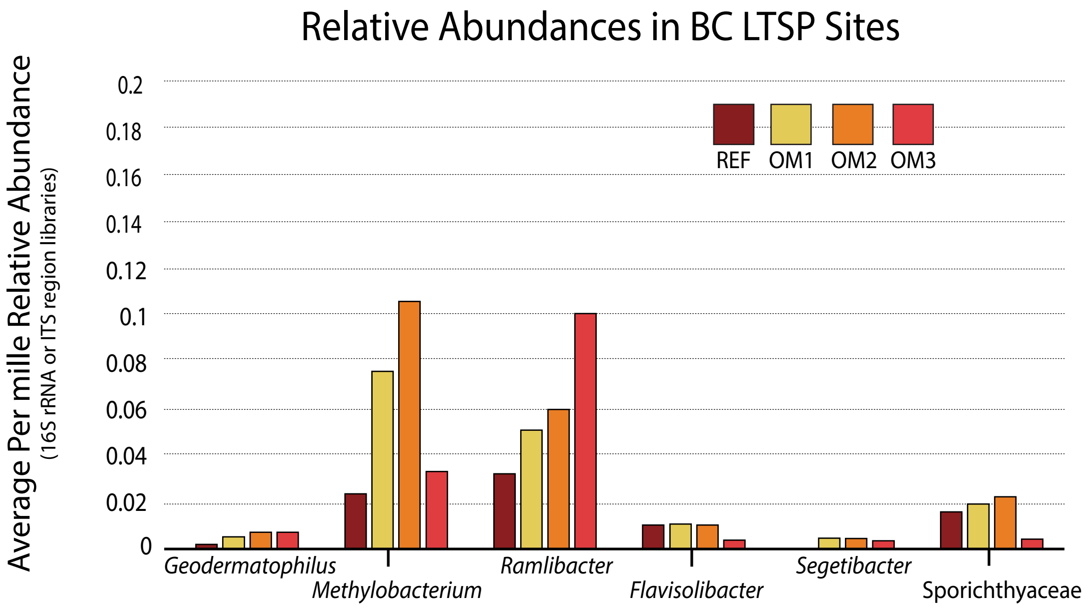

Supplement: Supplementary file 13 [file Image5.pdf]
