# Supplementary material for: Long-Term Enrichment of Stress-Tolerant Cellulolytic Soil Populations following Timber Harvesting Evidenced by Multi-Omic Stable Isotope Probing
Source: Front Microbiol. 2017 Apr 11;8:537. doi: 10.3389/fmicb.2017.00537 (PMC5386986; doi:10.3389/fmicb.2017.00537)

Figure S6. Non-metric multidimensional scaling of ITS pyrotag libraries based on Bray-Curtis dissimilarities. Ovals indicate the distribution of samples (grey crosses) that clustered according to ^{13}C-enrichment and soil layer. Coloured circles represent the ordination of fungal classes of greater than 0.15% overall relative abundance and are scaled to their normalized abundances in ^{12}C- (pink) and ^{13}C-libraries (blue).

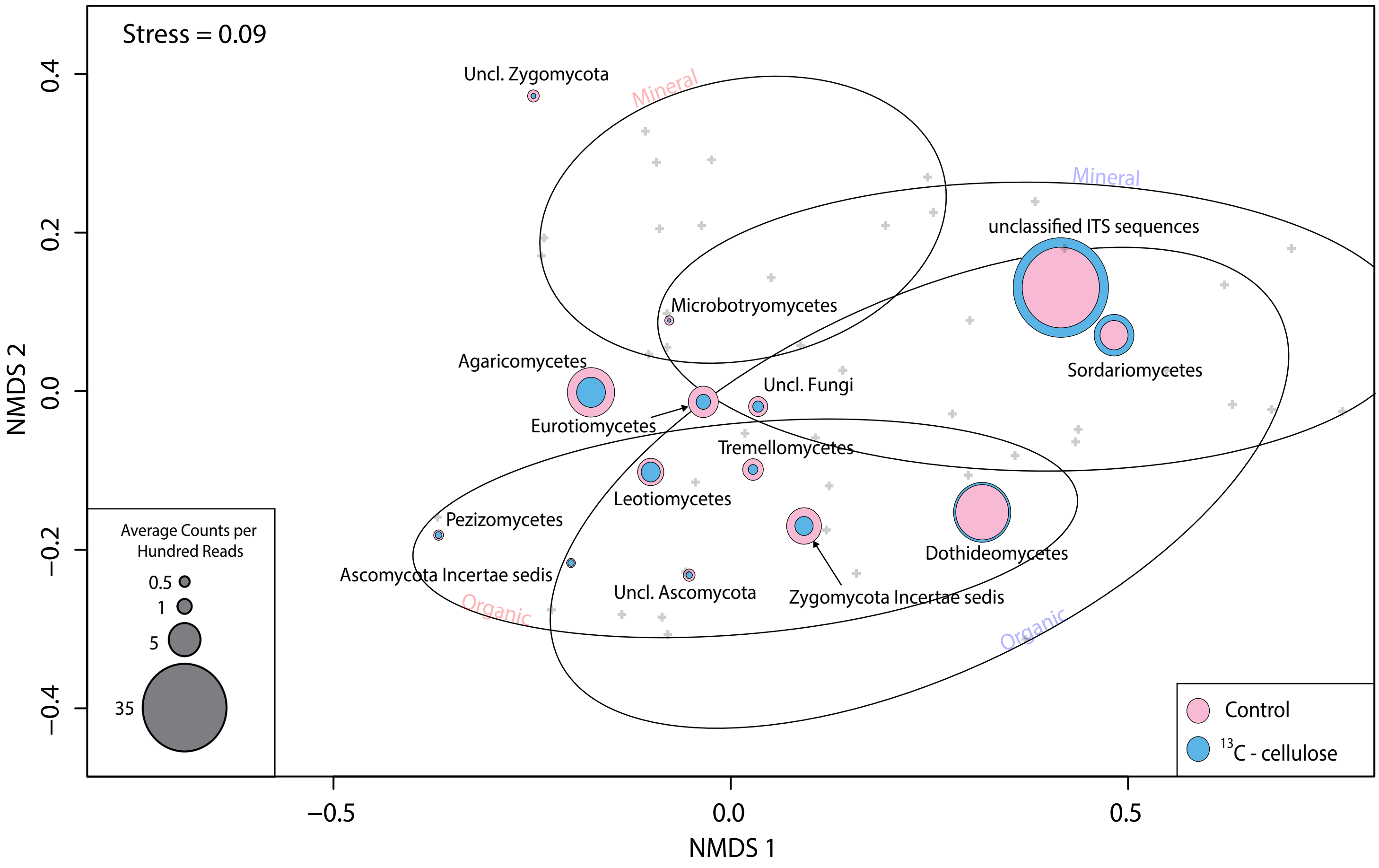

Supplement: Supplementary file 14 [file Image6.pdf]
