# Supplementary material for: Long-Term Enrichment of Stress-Tolerant Cellulolytic Soil Populations following Timber Harvesting Evidenced by Multi-Omic Stable Isotope Probing
Source: Front Microbiol. 2017 Apr 11;8:537. doi: 10.3389/fmicb.2017.00537 (PMC5386986; doi:10.3389/fmicb.2017.00537)

Figure S7. Lowest-common ancestor classification of all unassembled metagenomic reads at the phylum level. Phyla with less than 0.5% of the total were excluded.

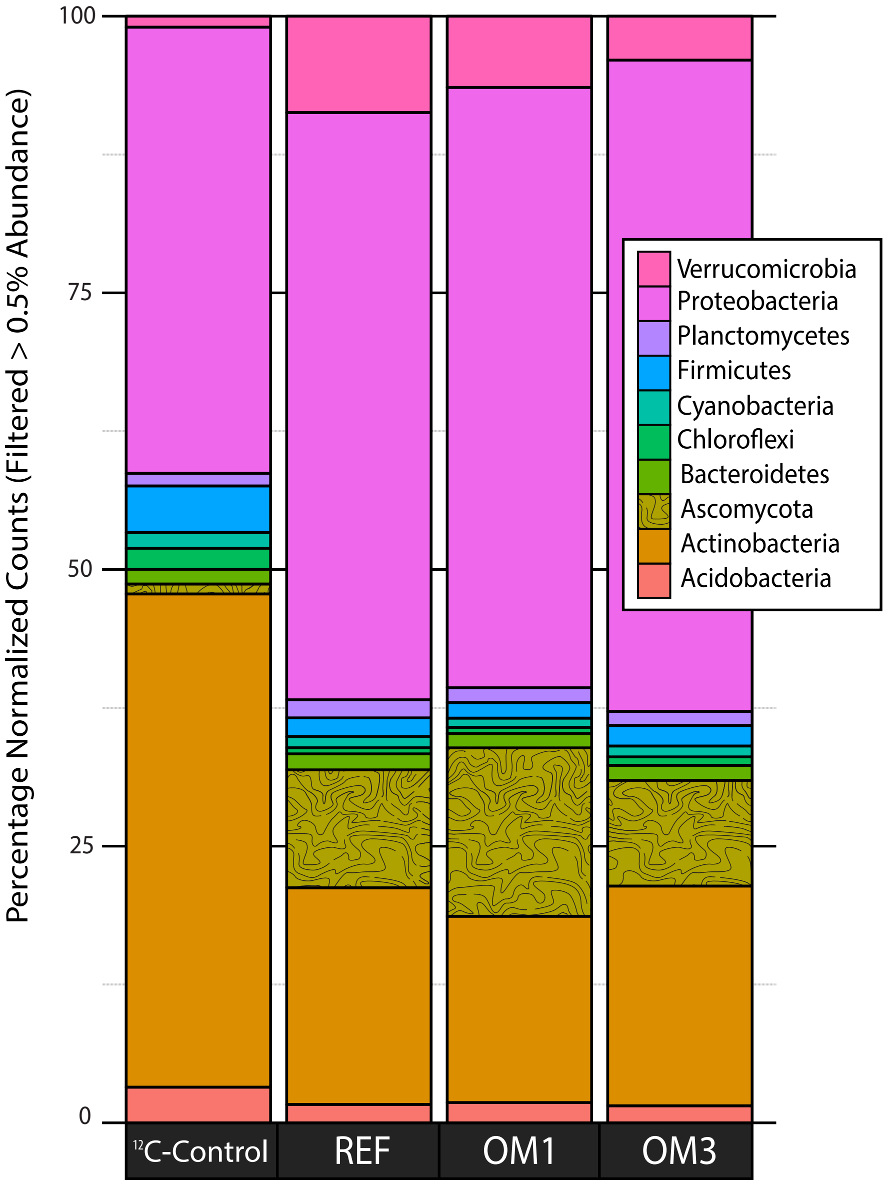

Supplement: Supplementary file 15 [file Image7.pdf]
